# Competing HB acceptors: an extensive NMR investigations corroborated by single crystal XRD and DFT calculations†

**DOI:** 10.1039/d1ra02538d

**Published:** 2021-04-22

**Authors:** Surbhi Tiwari, Neeru Arya, Sandeep Kumar Mishra, N. Suryaprakash

**Affiliations:** NMR Research Centre and Solid State and Structural Chemistry Unit, Indian Institute of Science Bangalore 560012 India nsp@iisc.ac.in suryaprakash1703@gmail.com +91 80 23601550 +91 80 23607344 +91 80 22933300 +91 98 45124802; Department of Physics and NMR Research Centre, Indian Institute of Science Education and Research Pune 411008 India

## Abstract

A series of *N*-benzoylanthranilamide derivatives have been synthesized with the substitution of competitive HB acceptors and investigated by NMR spectroscopy and single crystal XRD. The interesting rivalry for HB acceptance between <svg xmlns="http://www.w3.org/2000/svg" version="1.0" width="10.400000pt" height="16.000000pt" viewBox="0 0 10.400000 16.000000" preserveAspectRatio="xMidYMid meet"><metadata> Created by potrace 1.16, written by Peter Selinger 2001-2019 </metadata><g transform="translate(1.000000,15.000000) scale(0.011667,-0.011667)" fill="currentColor" stroke="none"><path d="M80 1160 l0 -40 40 0 40 0 0 -40 0 -40 40 0 40 0 0 -40 0 -40 40 0 40 0 0 -40 0 -40 40 0 40 0 0 -40 0 -40 40 0 40 0 0 -40 0 -40 40 0 40 0 0 -40 0 -40 40 0 40 0 0 80 0 80 -40 0 -40 0 0 40 0 40 -40 0 -40 0 0 40 0 40 -40 0 -40 0 0 40 0 40 -40 0 -40 0 0 40 0 40 -40 0 -40 0 0 40 0 40 -80 0 -80 0 0 -40z M560 520 l0 -40 -40 0 -40 0 0 -40 0 -40 -40 0 -40 0 0 -40 0 -40 -40 0 -40 0 0 -40 0 -40 -40 0 -40 0 0 -40 0 -40 -40 0 -40 0 0 -40 0 -40 -40 0 -40 0 0 -40 0 -40 80 0 80 0 0 40 0 40 40 0 40 0 0 40 0 40 40 0 40 0 0 40 0 40 40 0 40 0 0 40 0 40 40 0 40 0 0 40 0 40 40 0 40 0 0 80 0 80 -40 0 -40 0 0 -40z"/></g></svg> C=O and X (F or OMe) is observed in the investigated molecules which leads to an unusual increase in the electron density at the site of one of the NH protons, reflecting in the high field resonance in the ^1^H NMR spectrum. The NMR experimental findings and single crystal XRD are further reinforced by the DFT studies.

## Abstract 2

A series of *N*-benzoylanthranilamide derivatives have been synthesized with the substitution of competitive HB acceptors and investigated by NMR spectroscopy and single crystal XRD.

## Graphical abstract

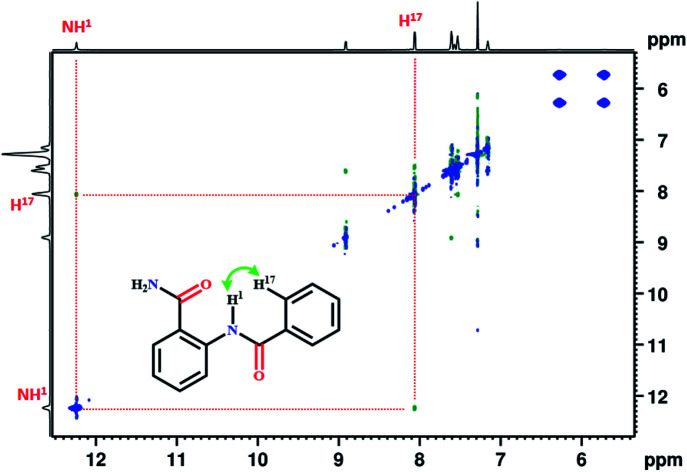

## Introduction

The hydrogen bond (HB) plays a key role in stabilizing the three-dimensional structures of many organic and biomolecules and has tremendous influence in chemistry, biology, drug design, *etc.*^1–3^ The HB can exist between an H atom covalently bonded to a donor atom (D) and acceptor atom(s) (A), where both D and A should be more electronegative than H.^4^ Based on the number of acceptor atoms the HB could be two-centered, or three-centered (bifurcated). The bifurcated HB can either be of (A⋯H⋯A) or (H⋯A⋯H) type.^5–9^ These HBs can be inter- or intra-molecular or a mixture of both types. The existence of inter- and/or intra-molecular HB may administer the architecture of various natural and synthetic compounds. The selective introduction of HBs may lead to the desired conformation of a molecule.^10^ The strength of any HB is directly related to the electronegativity of acceptor atom(s)^10^ and also depends on geometrical parameters, such as, the angle and the distance between H and acceptor atom. Owing to the electronegativity of N and O atoms, the strong HBs of N–H⋯O, O–H⋯O, and O–H⋯N motifs are usually encountered.^11^ The substantial fraction of the commercially available pharmaceutical drugs possess the fluorine atom(s) which alter their physical properties and improves the binding affinity with the target molecules through HB(s).^12–16^ Despite being the most electronegative atom,^17^ the participation of organic fluorine in the HB has been extensively debated. Earlier it was believed that organic fluorine hardly participates in the intramolecular HB.^18–22^ Nonetheless, a number of recent reports established the existence of intramolecular HB with the participation of fluorine attached to the carbon atom. The recent report also states that, “it is now difficult to doubt the existence of hydrogen bonds involving organic fluorine”.^19^

Among many available analytical techniques, the NMR spectroscopy has been proved to be the most valuable one in the study of HB. The change in chemical shift upon dilution with the solvents of different polarities, variable temperature studies, 2D HOESY and 2D HSQC experiments, clearly ascertain the presence or the absence of HB.^23,24^ The participation of fluorine in the HB is also evidenced by the detection of ^1h^*J*_FH_, mediated through non-covalent bond.^25^ It has been reported that the couplings between F and H separated by 5 covalent bonds (^5^*J*_FH_) is always less than 1 Hz. The detection of the significantly large coupling strength between ^19^F and ^1^H has been attributed to be HB mediated.^26–28^ There are several examples of detection of direct through space couplings between many homo- and hetero-nuclear spins, such as *J*_HF_, *J*_FF_*J*_PF_, and *J*_PP_, and between many other NMR active nuclei, and many reports have discussed the mechanism and strengths of such through-space interactions.^29^ The detection of *J*_FH_ has also been extensively debated as, whether it arises because of hydrogen bond or due to the overlap of electronic clouds.^29–33^ Some studies also attributed the term “through-space” and evidenced that the spin polarization could be transferred between the two nuclei, H and F, *via* hydrogen bonds.^34,35^

**Scheme 1:**
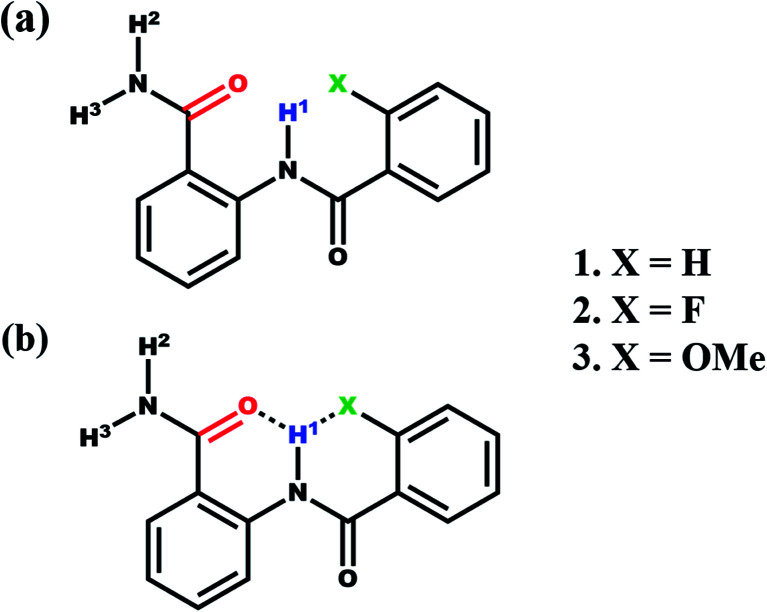
(a) The structural frameworks of *N*-benzoylanthranilamide and its derivatives along with the numbering of H atoms involved in HB; (b) illustration of the possible formation of a bifurcated HB.

The anthranilamide derivatives are pharmacologically important and known for their applications as antibacterial,^36,37^ antiviral,^38^ anticoagulants agents^39^ and also serve as a potent inhibitor of human factor Xa.^40,41^ Consequently, a series of *N*-benzoylanthranilamide were synthesized with *ortho* substitution at the benzoyl ring where one H, one donor (N) and two H acceptors (O and X = F, OMe) are present which satisfies the requirement of bifurcated (three-centered) intramolecular HB (Scheme 1). All these molecules were characterized and subjected to investigations by the utility of NMR experiments to ascertain the presence or absence of HBs. The basic structural frameworks of the molecules are reported in Scheme 1.

## Results and discussions

**Fig. 1:**
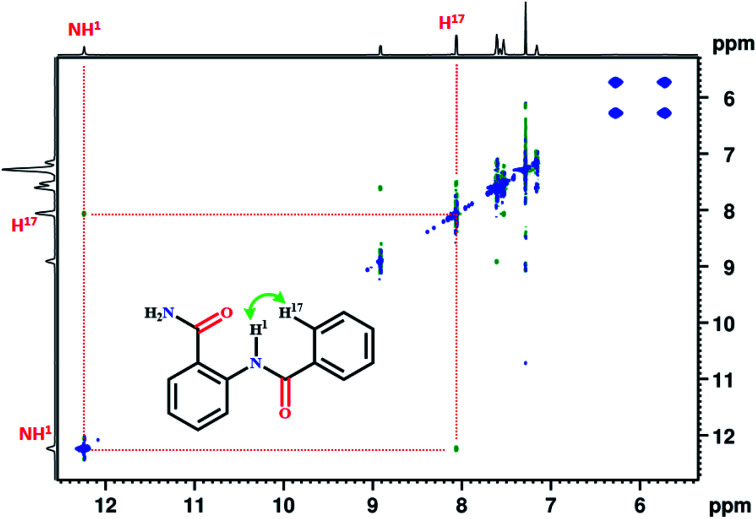
800 MHz 2D ^1^H–^1^H NOESY spectrum of molecule 1 in the solvent CDCl_3_ at 298 K.

Generally, the amide protons resonate between 5–9 ppm in the ^1^H NMR spectrum, and the formation of hydrogen bond leads to significant downfield shift. In the present study, the NH^1^ proton of molecule 1, resonated at 12.22 ppm. The extensive deshielding of this proton may be attributed to the intramolecular HB between NH^1^ proton and the carbonyl oxygen (C=O) of amide group. The 2D ^1^H–^1^H NOESY establishes the spatial proximity between two spins and aids in arriving at the favorable conformation of the molecule. The detection of correlation peak in the NOESY spectrum of molecule 1 (Fig. 1), corroborates the spatial proximity between the proton of NH^1^ and H^17^.

In molecule 2, the fluorine atom is presumed to be involved in an additional intramolecular HB with NH^1^, rendering the bifurcation. This assumption is strengthened by the detection of strong correlation peak between ^19^F and NH^1^ proton (Fig. S14†) in the 2D ^19^F–^1^H HOESY (Heteronuclear Overhauser Effect SpectroscopY) spectrum. Hence these results direct towards the existence of bifurcated HB in the investigated systems.

### Differentiating inter- and intra-molecular HB

**Fig. 2:**
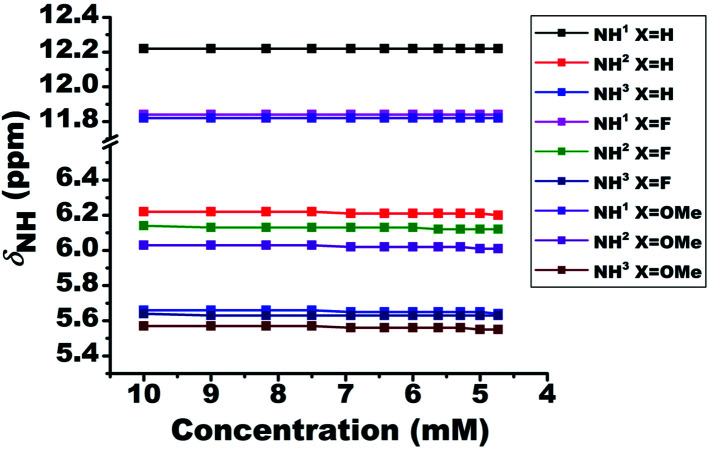
Variation in the chemical shifts of NH proton as a function of the concentration of solvent CDCl_3_, for the molecules 1–3. The initial concentration was taken as 10 mM in 450 ml of solvent at 298 K. The NH protons and the molecules are identified by numbers and different colour codes given in the inset.

To validate the intermolecular or intramolecular HB and to ascertain the effect of monomeric water on HB^42^ if any, the dilution study^23^ using a non-polar solvent CDCl_3_ was carried out. The dilution results in the dispersion of the molecules and consequently a substantial change in the chemical shift of protons when the interactions are of intermolecular type. However, the chemical shift remains invariant when the interaction is intramolecular. The plot of NH^1^ chemical shifts as a function of dilution with solvent CDCl_3_, for all the molecules, is given in Fig. 2. Although the solute concentration was not diluted to a large extent, the invariance of chemical shifts of NH^1^ proton when diluted to half its value, safely discards the possibility of any intermolecular interactions and confirming the existence of intramolecular HBs. However, the NH^2^ and NH^3^ protons exhibited a slight shift towards the shielded region on dilution. The negligible change in the chemical shift of residual water peak (1.54 ppm) ascertains the trifling effect of monomeric water^8,23,42^ on the intramolecular HB.

### Relative strengths of HB

**Fig. 3:**
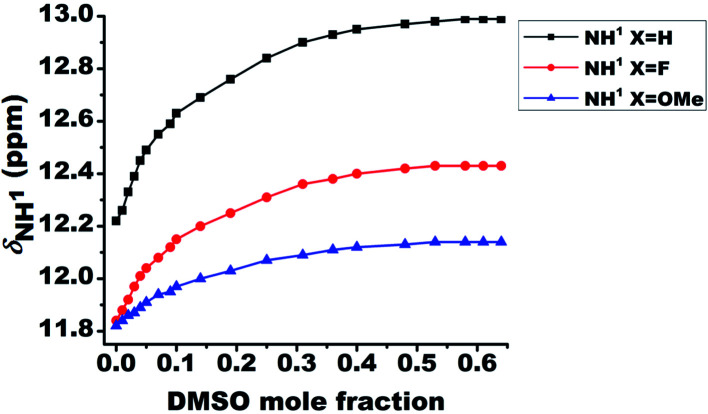
Change in the chemical shift of NH^1^ protons for the molecules 1–3 on addition of 0.65 mole fraction of DMSO-d_6_ at 298 K. The initial concentration taken was 10 mM in 450 ml of CDCl_3_ and DMSO-d_6_ was systematically added to it.

The relative strengths of intramolecular HB interactions can be estimated by the titration study with a highly polar solvent dimethyl sulfoxide (DMSO).^43^ Due to the high affinity towards HB acceptance, the solvent DMSO is capable of rupturing a variety of inter- or intra-molecular HBs. Hence the titration with systematic addition of DMSO-d_6_ to the 10 mM solutions of the molecules 1–3 in CDCl_3_ were carried out and the variation in chemical shift of NH^1^ peak was monitored (Fig. 3). The severe overlap of the NH^2^ and NH^3^ resonances with the aromatic protons hindered the determination of the effect of DMSO on these peaks. On incremental addition of DMSO-d_6_, the chemical shift of NH^1^ proton was shifted to higher frequency region for all the molecules, which is attributed to the engagement of NH^1^ proton in the intermolecular HB with DMSO (Fig. 3).^8,44,46^ The deshielding in the NH^1^ proton chemical shift is inversely proportional to the strength of intramolecular HB in such systems^44,46^ because the intramolecular HB minimizes the accessibility of sites for DMSO around the acidic proton. The small change in chemical shifts upon DMSO-d_6_ addition could possibly be attributed to the favorable near planar geometry of these molecules which also limits the accessibility of the sites for the association of DMSO by creating hindrance. The change in the NH^1^ chemical shifts for molecules 1–3 on addition of 0.65 mole fraction of DMSO are reported in Table 1.

**Table 1:** The difference in NH^1^ chemical shifts of the molecules 1–3 with the addition of DMSO in mole fractions

Molecule	Substituent (X)	*δ*_NH^1^_ (ppm)		Difference in chemical shift *δ*_NH^1^_ (ppm)
		DMSO-d_6_ (in mole fraction = 0)	DMSO-d_6_ (in mole fraction = 0.65)	
1	H	12.22	12.99	0.77
2	F	11.84	12.43	0.59
3	OMe	11.83	12.14	0.31

### Effect of temperature

**Fig. 4:**
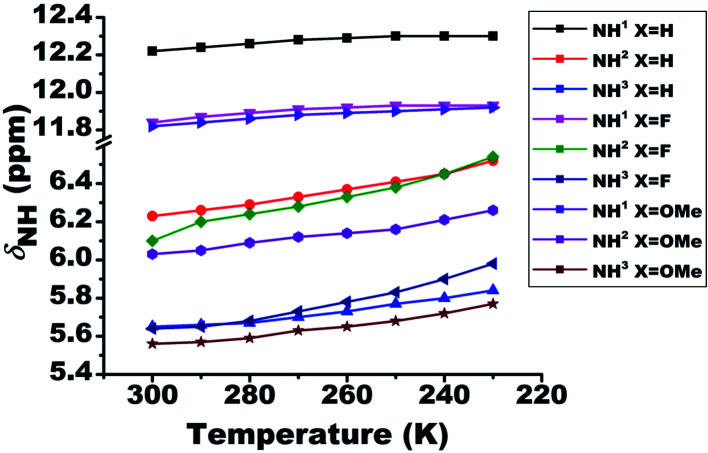
Effect of temperature on the chemical shifts of NH protons (*δ*_NH_) of molecules 1–3. The temperature was varied from 300 K to 230 K. The solution concentration in CDCl_3_, was maintained at 10 mM.

The lowering of temperature leads to the strengthening of HB, which results in the deshielding of the proton involved in the HB. On systematically varying the temperature from 300 K to 230 K the chemical shift of the NH^1^ proton moved towards the higher resonance frequency as a consequence of the strengthening of the intramolecular HB (Fig. 4). However, in the case of NH^2^ and NH^3^ protons, the shift towards the deshielded region (Fig. 4) is attributed to the decrease in the electron density on amide (>CO–NH_2_) group. Additionally, the deshielding in NH^2^ and NH^3^ proton chemical shifts also point towards the existence of HB. However, from the close inspection of the chemical structures, these deshielding can be attributed to intermolecular HBs between carbonyl oxygen and these protons at low temperature (Fig. 4), rather than intramolecular HB. This possibility was also inferred from the dilution studies with CDCl_3_ solvent (Fig. 2), which is now confirmed. The calculated amide temperature coefficient values (Δ*δ*_NH^1^_/Δ*T*) also reveal the small variation in the chemical shift of NH^1^ protons with temperature, *viz.*, from −0.3 to −1.3 ppb K^−1^ and are assimilated in Table S1.† These values, which is more positive than −0.4 ppb K^−1^, the HB predictivity is more than 85%.^47^

### Detection of ^1h^*J*_XH_

**Fig. 5:**
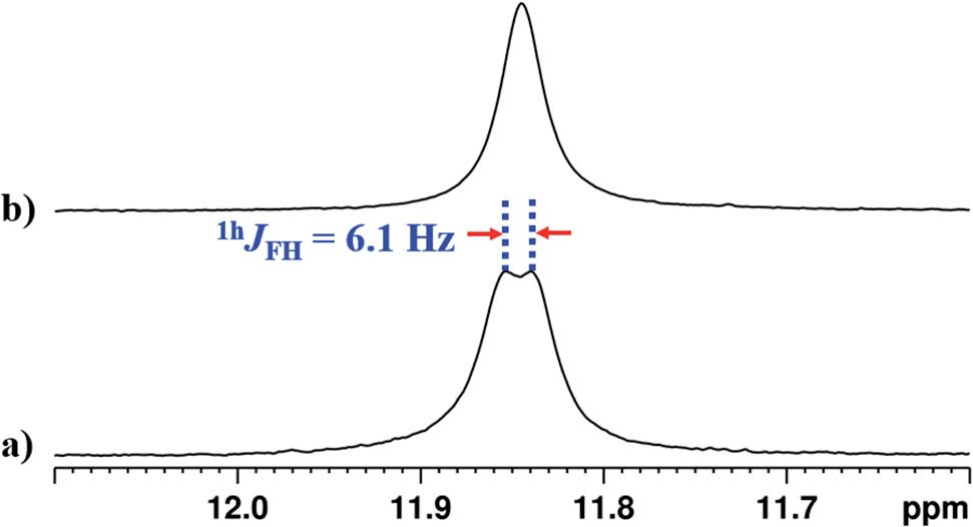
400 MHz ^1^H spectrum corresponding to proton NH^1^ of molecule 2 in the solvents CDCl_3_; (a) ^19^F coupled spectrum and (b) ^19^F decoupled spectrum.

The interaction of NH^1^ proton with the acceptor atom X can be reflected as *J* coupling in the 1D ^1^H NMR spectrum, if X is also an NMR active nucleus. The NH^1^ proton in the molecule 2 appeared as a doublet with the frequency separation of 6.1 Hz. The 1D ^1^H{^19^F} NMR experiment on molecule 2 confirmed the interaction between ^1^H and ^19^F, where NH^1^ proton appeared as a singlet (Fig. 5b). In the present study, the observed ^1h^*J*_FH_ value of 6.1 Hz (Fig. 5a) is small compared to the previous reports,^8,44,45^ and can be attributed to the presence of another strong HB acceptor (C=O group of amide) which pulls the NH^1^ proton far from the F atom, which resulted in the decreased F⋯HN strength. To extract the hidden couplings, if any, the 1D ^1^H{^14^N} and 2D ^15^N–^1^H coupled HSQC experiments at the natural abundance of ^15^N were carried out for molecule 2, and the spectra are reported in Fig. S10 and S17 respectively in the ESI.† These spectra yielded ^1h^*J*_FH_ of 7.68 Hz, which implied that the observed doublet of 6.1 Hz for NH^1^ proton in Fig. 5a, also had the contribution from the unresolved ^1^*J*_^14^N–^1^H_ and the additional broadening arose due to the ^14^N quadrupole relaxation.

**Fig. 6:**
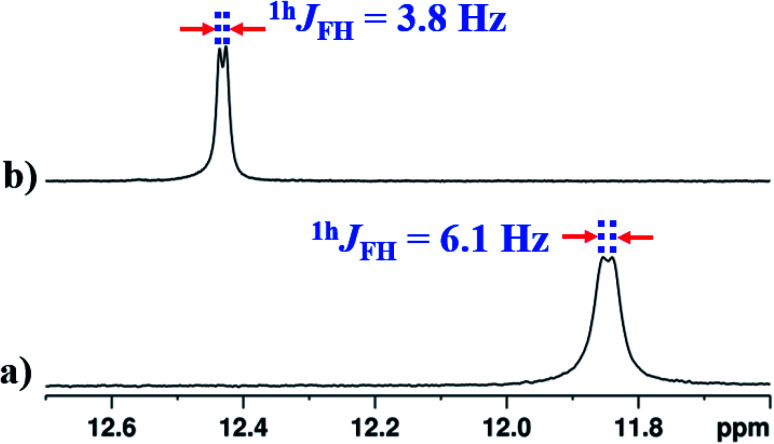
400 MHz of ^1^H NMR spectrum of molecule 2, at 298 K, corresponding to NH^1^ proton; (a) in CDCl_3_ solvent; (b) in DMSO-d_6_ solvent.

In the solvent DMSO-d_6_, the observed ^1h^*J*_FH_ was only 3.8 Hz in both 1D ^1^H and 1D ^1^H{^14^N} spectra (Fig. 6(b) and S13†). However, in many earlier reports, this coupling completely vanished^45,46,48,49^ in DMSO except in molecules where the structural restraint resists the complete breaking of HB by DMSO.^26,44^ The retention of the ^1h^*J*_FH_ for molecule 2 in the DMSO-d_6_ is due to the favorable *cis* geometry (Fig. 1), which prevents the complete solvation of HB. The molecules tend to surround the NH^1^ proton causing the steric hindrance whereby the distance between NH^1^ proton and fluorine atom increases, leading to the decrease in ^1h^*J*_FH_. However, in the earlier reports, the *cis* form of the structure stabilizes in a non-polar solvent by the influence of intramolecular HB and the molecules attain the lowest energy structure by rupturing the HB in DMSO which is different from the *cis* form, resulting in a complete nullification of ^1h^*J*_FH_.

### Variation in ^1h^*J*_FH_

**Fig. 7:**
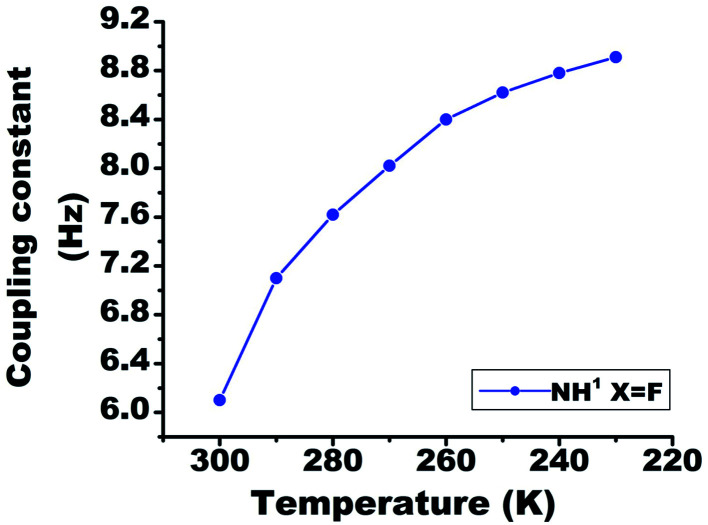
The temperature dependent variation in the ^1h^*J*_FH_ in molecule 2, in the range 300 K to 230 K. The solute concentration was taken 10 mM in the solvent CDCl_3_.

On lowering the temperature from 300 K to 230 K, the value of ^1h^*J*_FH_ in molecule 2 changed from 6.1 Hz to 8.9 Hz (Fig. 7). The covalent bond mediated scalar coupling remains practically invariant, while the HB mediated coupling varies with changing the distance between H and acceptor atom.^23^ Therefore, this significant variation in the ^1h^*J*_FH_ for molecule 2, is attributed to the coupling mediated through HB.

### Unusual chemical shift value of NH^1^ proton

**Fig. 8:**
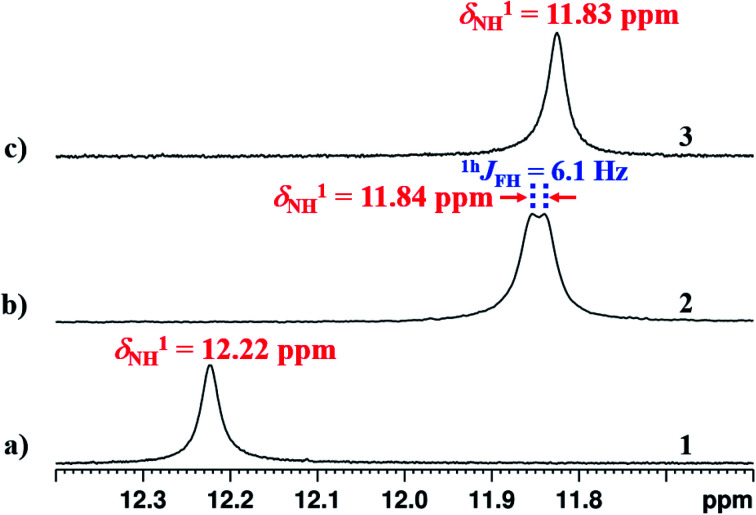
400 MHz ^1^H NMR spectra in CDCl_3_ solvent at 298 K, corresponding to the NH^1^ proton region of; (a) molecule 1; (b) molecule 2; and (c) molecule 3.

Conventionally, the presence of HB or the introduction of additional HB(s) results in the deshielding of proton in the ^1^H-NMR spectrum. Contrary to the expectation, the NH^1^ proton is observed to be more shielded (Fig. 8(b) and (c)) on substitution of X at the *ortho* position of benzoyl ring (in molecules 2 and 3). This is due to the fact that the NH^1^ proton is involved in a strong HB with the C=O oxygen of amide group. Furthermore, the substitution of F or OMe increases the electron density on the NH^1^ proton by stabilizing an equilibrium between two hydrogen acceptors resulting in the shielding of proton.^49^ Another possible reason could be the weakening of strong two-center C=O⋯H HB during the competition with another HB acceptor.

**Fig. 9:**
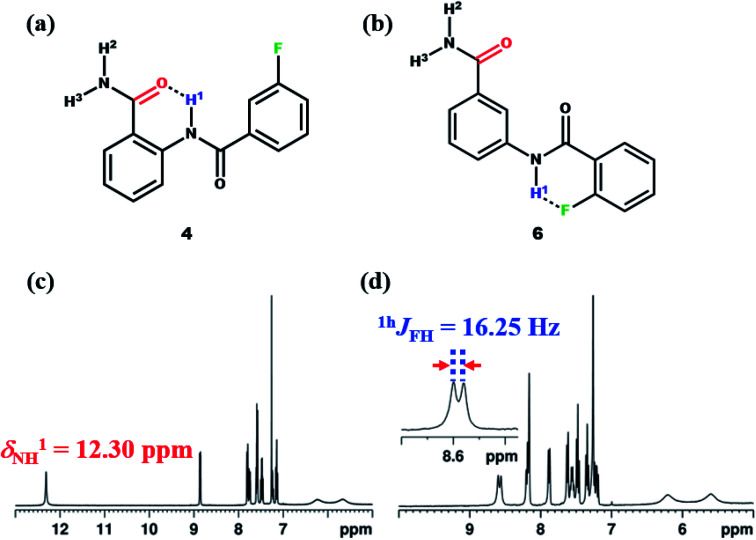
The chemical structures of; (a) molecule 4, and (b) molecule 6; (c and d) the corresponding 400 MHz ^1^H NMR spectra in the CDCl_3_ solvent recorded at 298 K.

To derive more insight, additionally the molecules 4–7 were synthesized (Fig. 9a, S31, 9b and S36). When the F in the molecule 4 was displaced from *ortho* to *meta* position on the benzoyl ring with respect to NH^1^, the value of chemical shift of NH^1^ was observed to be similar to that of molecule 1 (Fig. 9c). Also considering the electronic effect, when the F was displaced to *para* position in molecule 5 (ESI, Fig. S31†), the observed chemical shift value was similar to those detected in molecules 1 and 4. Subsequently, when the amide group of the molecule 2 was displaced from *ortho* to *meta* position (molecule 6), the NH^1^ proton exhibited a doublet with a separation of 16.25 Hz (Fig. 9d), whose value is similar to those observed for other reported molecules.^45,46,48–52^

All these in-depth NMR studies leads to the conclusion that the N–H⋯X interactions are influenced by strong C=O⋯H HB, which is also reflected in the shielding of NH^1^ proton and reduced value of ^1h^*J*_FH_ in the molecule 2. These narrations suggest that the competitive equilibrium between N–H⋯X and C=O⋯H–N type HBs debilitate with each other and is the possible reason for the shielding of NH^1^ proton in the molecules 2 and 3 compared to 1. The aforesaid facts are also corroborated by findings from the single crystal XRD and theoretical calculations discussed in the forthcoming part of this manuscript. A good single crystal for molecule 2 was obtained, and unfortunately, we failed in our efforts to crystallize other molecules, and thus XRD study is restricted only to molecule 2.

### Single crystal X-ray diffraction (XRD) studies

**Fig. 10:**
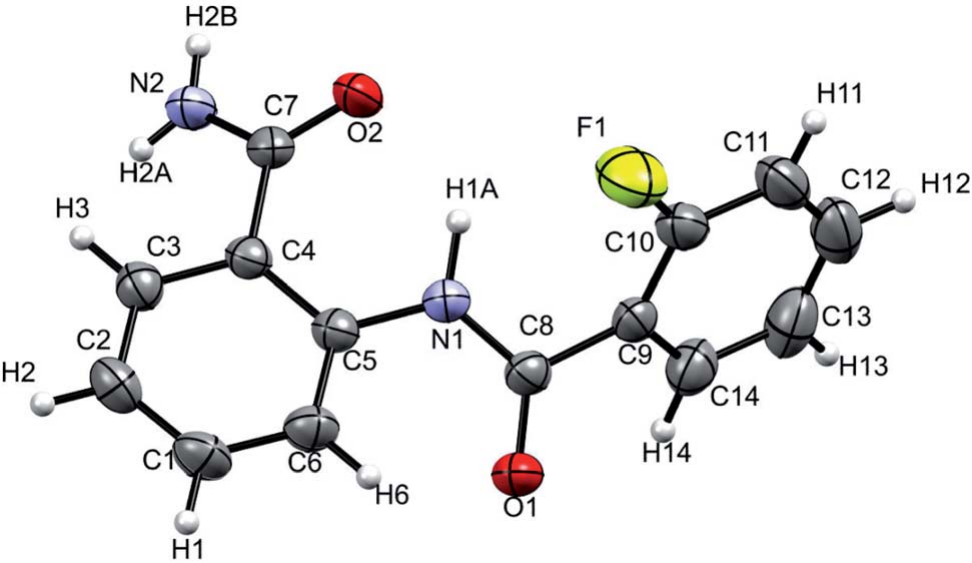
Single-crystal XRD structure of molecule 2 at 298 K with ORTEP view with 50% probability level (CCDC number: 2049223†).

The XRD is another powerful technique for the investigation of HB, where the linearity in the bond angle *i.e.* D–H⋯A of 180° and closer to this value validates the presence of stronger HB.^53,54^ The distance between the H and the acceptor atom between 1.2–1.5 Å also indicates a strong HB, whereas the distance of 1.5–2.2 Å suggests the HB of moderate strength and the value > 2.2 Å establishes a relatively weak HB.^53^ The XRD structure of the molecule 2 is reported in Fig. 10.

The NH^1^⋯F distance and the N–H^1^⋯F angle were determined as 2.310 Å and 110° respectively, which corresponds to a weak HB in the solid state. The results derived from the single crystal XRD are assimilated in Table 2 and the experimental details are provided in ESI (Table S2†).

**Table 2:** The DFT computed structural parameters and single crystal XRD studies. All the bond distances (*d*) are expressed in Å; bond angles (*φ*) and torsion angles (*Φ*) are in degrees

Parameter	DFT							XRD
	Molecule							Molecule
	1	2	3	4	5	6	7	2
*d*_N–H^1^_	1.018	1.019	1.018	1.019	1.018	1.009	1.018	0.965
*d*_O⋯^1^HN_	1.821	1.847	2.013	1.798	1.808	—	1.829	1.974
*d*_NH^1^⋯F/OMe_	—	2.310	1.938	—	—	1.895	—	2.322
*φ*_NH^1^O_	139.2	138.3	130.1	139.94	139.71	—	138.60	139.2
*φ*_NH^1^F/NH^1^OMe_	—	110.0	128.4	—	—	137.34	—	109.3
*Φ* ^1^_N1C5C4C7_	−2.41	−6.30	−0.68	−2.41	−2.60	—	−2.43	−2.4(3)
*Φ* ^2^_N1C8C9C10_	−22.82	38.24	−15.025	21.86	18.31	2.24	−26.96	39.8(3)
Energy of HB (*E*_HB_) (kcal mol^−1^)	—	−2.823	1.283	—	—	2.902	—	—

### DFT computations

**Fig. 11:**
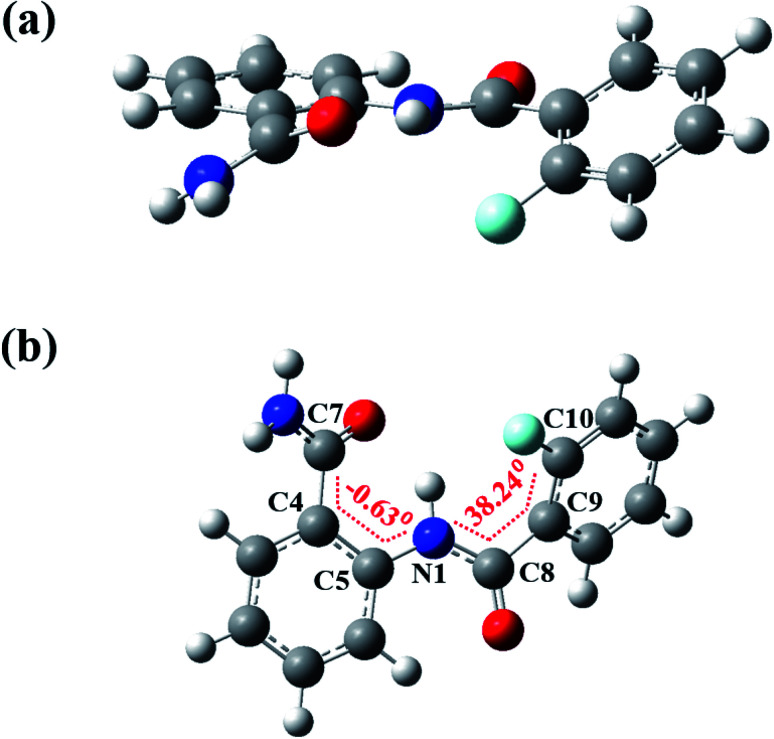
The DFT optimized spatial structure of molecule 2; (a) and (b) are two different projections.

The DFT based computations carried out also reinforces the NMR and XRD findings. The lowest energy structures were optimized using Gaussian09 suite with B3LYP/6-311+g(d,p) level of basis set using chloroform as the default solvent.^55^ The optimized structure of molecule 2 is reported in Fig. 11 and of molecules 1 and 3 in Fig. S40 and S41† respectively, and the computed structural parameters are provided in Table 2. The formation of three-center HB usually increases the D–H bond lengths with decrease in the H⋯X distance and D–H⋯A bond angle^4^ tending towards the planar geometry. Thus, the theoretical computations highlight the following aspects:

• The bond length (*d*_N–H^1^_) of molecule 2 (1.019 Å) is observed to be longer than the molecule 1 (1.018 Å) whereas the molecule 3 showed no significant change (1.018 Å).

• The distance between H and the X (X = F or OMe) was observed 2.310 Å and 1.938 Å in the molecules 2 and 3 respectively, suggesting the presence of a weak HB.

• The N–H^1^⋯O bond angles in molecules 1, 2 and 3 were determined to be, 139.2°, 138.3° and 130.1° respectively, whereas the N–H^1^⋯X (X = F or OMe) bond angles in molecules 2 and 3 were found to be 110° and 128.4°, respectively, suggesting the comparatively strong N–H^1^⋯O=C <svg xmlns="http://www.w3.org/2000/svg" version="1.0" width="10.400000pt" height="16.000000pt" viewBox="0 0 10.400000 16.000000" preserveAspectRatio="xMidYMid meet"><metadata> Created by potrace 1.16, written by Peter Selinger 2001-2019 </metadata><g transform="translate(1.000000,15.000000) scale(0.011667,-0.011667)" fill="currentColor" stroke="none"><path d="M480 1160 l0 -40 -40 0 -40 0 0 -40 0 -40 -40 0 -40 0 0 -40 0 -40 -40 0 -40 0 0 -40 0 -40 -40 0 -40 0 0 -40 0 -40 -40 0 -40 0 0 -80 0 -80 40 0 40 0 0 40 0 40 40 0 40 0 0 40 0 40 40 0 40 0 0 40 0 40 40 0 40 0 0 40 0 40 40 0 40 0 0 40 0 40 40 0 40 0 0 40 0 40 40 0 40 0 0 40 0 40 -80 0 -80 0 0 -40z M80 480 l0 -80 40 0 40 0 0 -40 0 -40 40 0 40 0 0 -40 0 -40 40 0 40 0 0 -40 0 -40 40 0 40 0 0 -40 0 -40 40 0 40 0 0 -40 0 -40 80 0 80 0 0 40 0 40 -40 0 -40 0 0 40 0 40 -40 0 -40 0 0 40 0 40 -40 0 -40 0 0 40 0 40 -40 0 -40 0 0 40 0 40 -40 0 -40 0 0 40 0 40 -40 0 -40 0 0 40 0 40 -40 0 -40 0 0 -80z"/></g></svg> HB than N–H^1^⋯X.

• The molecules 1–3 lacks the planarity which is also noticed on comparing the torsion angles (Table 2). In the molecule 2 the torsion angle (*Φ*^2^_N1C8C9C10_ = 38.24°) was found to be higher than in the molecule 1 (*Φ*^2^_N1C8C9C10_ = −22.82°), whereas in the molecule 3 it was observed much close to the planarity (*Φ*^2^_N1C8C9C10_ = −15.02°), which indicates the stronger interaction by methoxy group compared to the fluorine substituent.

• The energies of HBs (*E*_HB_) were calculated as −2.823 and −1.283 kcal mol^−1^ for molecules 2 and 3 respectively, showing agreement with the presence of weak HBs.

• The substitution of X (F or OMe) in the molecules 2 and 3 leads to the increase in the O⋯H distance in O⋯^1^H–N (*d*_O⋯H^1^_). All the O⋯H distances are reported in the Table 2.

The theoretically computed structural parameters of the molecules 1–3 are also compared with those of molecules 4–7 in the Table 2. The DFT optimized minimum energy structures of molecules 4–7 are reported in Fig. S42–S45 of ESI.† The displacement of X (F or OMe) from *ortho* to *meta* position in the molecules 4 and 7 resulted in the decrease in the O⋯H distances (*d*_O⋯H^1^_ = 1.798 Å and 1.829 Å, respectively) compared to the molecules 2 and 3 (*d*_O⋯H^1^_ = 1.847 Å and *d*_O⋯H^1^_ = 2.013 Å, respectively), indicating the strong O⋯H HB in the molecules 4 and 7. However, on shifting the amide group from *ortho* to *meta* position in the molecule 6, the strength of observed H⋯F HB increases and displayed the planarity in the structure with a torsion angle of 2.24° which is significantly less compared to that of molecule 2 (*Φ*^2^_N1C8C9C10_ = 38.24°) (ESI, Fig. S44†). The chemical shift of NH^1^ (*δ*_NH^1^_) computed from the DFT optimized minimum energy structures and the experimently observed chemical shifts of molecules 1–7 are reported in Table S3.† The results obtained from DFT and single crystal XRD for molecule 2 are compared in Table 2.

## Conclusions

The presence of weak HB in the *N*-bezoylanthranilamide and its derivatives is substantiated by one- and two-dimensional NMR experimental investigations. The strong correlated peak in the 2D NOESY (molecule 1) and 2D HOESY (molecule 2) spectra ascertain the spatial propinquity between NH^1^ and X (F or methoxy), leading to the existence of bifurcated HB. The doublet for NH^1^ proton provided clear evidence for the HB between ^19^F and NH^1^ proton. The residual ^1h^*J*_FH_ (∼60%) in the high polarity solvent DMSO-d_6_ and 2D ^1^H–^1^H NOESY experiments confirmed the existence of favorable *cis* conformers for the investigated molecules. The rivalry between N–H⋯X and C=O⋯H–N types of HBs is perceived as unusual shielding in NH^1^ resonance frequency of molecules 2 and 3 and comparatively small ^1h^*J*_FH_ coupling in the molecule 2. The NMR experimental findings are strongly supported by the single crystal XRD and DFT computational studies.

## Experimental

All the NMR spectra were recorded using 400 MHz and 800 MHz spectrometers at 298 K, except for the variable temperature studies. The TMS was used as an internal reference to measure the proton chemical shifts. The synthesized molecules were characterized by electron spray ionization mass spectrometry (ESI-HRMS) and various one- and two-dimensional NMR techniques. The commercially available chemicals, including deuterated solvents, were purchased and used as received. The XRD data was collected on a diffractometer with Mo K_α_ radiation. The structure was solved by direct methods using SHELXS97 (ref. 56) and refined in the spherical atom approximation (based on *F*^2^) by SHELXL97 (ref. 56) using the WinGX suite (ref. 57).

## General synthesis of molecules 1 to 7

The 1 equivalent of benzoyl chloride (500 mg, 3.67 mmol) of interest and pyridine (290.29 mg, 3.67 mmol) was added dropwise to the 1.09 equivalent of amino benzamide (4.003 mmol) of interest solution in 15 ml of chloroform at 0 °C. After that, the ice bath is removed, and the reaction mixture was stirred at room temperature for 1 hour. A precipitate obtained was filtered and washed with a copious amount of water. The trace of pyridine was evaporated by adding toluene solvent. The formation of *N*-benzoylanthranilamide and its derivatives was characterized by electron spray ionization mass spectrometry (ESI-HRMS) and using NMR techniques.

## Conflicts of interest

Authors declare no conflict of interest.

## Supplementary Material

RA-011-D1RA02538D-s001

RA-011-D1RA02538D-s002
