# Supplementary material for: Competing HB acceptors: an extensive NMR investigations corroborated by single crystal XRD and DFT calculations
Source: RSC Adv. 2021 Apr 22;11(25):15195–202. doi: 10.1039/d1ra02538d (PMC8698242; doi:10.1039/d1ra02538d)
Supplement: RA-011-D1RA02538D-s001 [file RA-011-D1RA02538D-s001.pdf]

Electronic Supplementary Material (ESI) for RSC Advances.
This journal is © The Royal Society of Chemistry 2021

Supporting Information

Competing HB Acceptors: An extensive NMR Investigations Corroborated by Single Crystal XRD and DFT Calculations

Surbhi Tiwari[a], Neeru Arya[a], Sandeep Kumar Mishra[b] and N. Suryaprakash*[a]

[a]NMR Research Centre and Solid State and Structural Chemistry Unit, Indian Institute of Science, Bangalore 560012, India
[b]Department of Physics and NMR Research Centre, Indian Institute of Science Education and Research, Pune 411008, India

E-mail: nsp@iisc.ac.in; suryaprakash1703@gmail.com ; Fax: +91 8023601550; Tel: +91 8023607344; +91 80 22933300; +919845124802 (Cell)

Table of Contents

Figure S1: ^{1}H NMR spectrum of molecule **1**, acquired in 400 MHz spectrometer in the solvent $CDCl_3$ at 298K.

Figure S2: ^{1}H NMR spectrum of molecule **1**, acquired in 400 MHz spectrometer in the solvent DMSO at 298K.

Figure S3: ^{13}C NMR spectrum of molecule **1**, acquired in 800 MHz spectrometer in the solvent $CDCl_3$ at 298K.

Figure S4: 2D ^{1}H-^{13}C HSQC NMR spectrum of molecule **1**, acquired in 800 MHz spectrometer in the solvent $CDCl_3$ at 298K.

Figure S5: 2D ^{1}H-^{15}N coupled HSQC NMR spectrum of molecule **1** of NH^1 region, acquired in 400 MHz spectrometer in the solvent $CDCl_3$ at 298K.

Figure S6: 2D ^{1}H-^{15}N decoupled HSQC NMR spectrum of molecule **1** of NH^1 region, acquired in 400 MHz spectrometer in the solvent $CDCl_3$ at 298K.

Figure S7: HRMS (ESI) $[M+Na]^+$ spectrum of molecule **1**.

Figure S8: ^{1}H NMR spectrum of molecule **2**, acquired in 400 MHz spectrometer in the solvent $CDCl_3$ at 298K.

Figure S9: $^{1}H\{^{19}F\}$ NMR spectrum of molecule **2**, acquired in 400 MHz spectrometer in the solvent $CDCl_3$ at 298K.

Figure S10: $^{1}H\{^{14}N\}$ NMR spectrum of molecule **2**, acquired in 400 MHz spectrometer in the solvent $CDCl_3$ at 298K.

Figure S11: ^{1}H NMR spectrum of molecule **2**, acquired in 400 MHz spectrometer in the solvent DMSO at 298K.

Figure S12: $^{1}H\{^{19}F\}$ NMR spectrum of molecule **2**, acquired in 400 MHz spectrometer in the solvent DMSO at 298K.

Figure S13: $^{1}H\{^{14}N\}$ NMR spectrum of molecule **2**, acquired in 400 MHz spectrometer in the solvent DMSO at 298K.

Figure S14: 400 MHz two dimensional ^{19}F-^{1}H HOESY spectrum of fluorine substituted molecule (molecule **2)** of NH^1 region in the solvent $CDCl_3$ at 298 K.

Figure S15: ^{13}C NMR spectrum of molecule **2**, acquired in 800 MHz spectrometer in the solvent $CDCl_3$ at 298K.

Figure S16: 2D ^{1}H-^{13}C HSQC NMR spectrum of molecule **2**, acquired in 800 MHz spectrometer in the solvent $CDCl_3$ at 298K.

Figure S17: 2D ^{1}H-^{15}N coupled HSQC NMR spectrum of molecule **2** of NH^1 region, acquired in 400 MHz spectrometer in the solvent $CDCl_3$ at 298K. The chemical structure of the molecule shows the couplings $^{1}J_{NH}$, $^{1h}J_{FH}$ and $^{2h}J_{FN}$ and are identified by arrows red, blue and black respectively.

Figure S18: 2D ^{1}H-^{15}N decoupled HSQC NMR spectrum of molecule **2** of NH^1 region, acquired in 400 MHz spectrometer in the solvent $CDCl_3$ at 298K.

Figure S19: HRMS (ESI) $[M+Na]^+$ spectrum of molecule **2**.

Figure S20: ^{1}H NMR spectrum of molecule **3**, acquired in 400 MHz spectrometer in the solvent $CDCl_3$ at 298K.

Figure S21: ^{1}H NMR spectrum of molecule **3**, acquired in 400 MHz spectrometer in the solvent DMSO at 298K.

Figure S22: ^{13}C NMR spectrum of molecule **3**, acquired in 800 MHz spectrometer in the solvent $CDCl_3$ at 298K.

Figure S23: 2D ^{1}H-^{13}C HSQC NMR spectrum of molecule **3**, acquired in 800 MHz spectrometer in the solvent $CDCl_3$ at 298K.

Figure S24: 2D ^{1}H-^{15}N coupled HSQC NMR spectrum of molecule **3** of NH^1 region, acquired in 400 MHz spectrometer in the solvent $CDCl_3$ at 298K.

Figure S25: 2D ^{1}H-^{15}N decoupled HSQC NMR spectrum of molecule **3** of NH^1 region, acquired in 400 MHz spectrometer in the solvent $CDCl_3$ at 298K.

Figure S26: HRMS (ESI) $[M+Na]^+$ spectrum of molecule **3**.

Figure S27: ^{1}H NMR spectrum of molecule **4**, acquired in 400 MHz spectrometer in the solvent $CDCl_3$ at 298K.

Figure S28: ^{13}C NMR spectrum of molecule **4**, acquired in 800 MHz spectrometer in the solvent $CDCl_3$ at 298K.

Figure S29: 2D ^{1}H-^{13}C HSQC NMR spectrum of molecule **4**, acquired in 800 MHz spectrometer in the solvent $CDCl_3$ at 298K.

Figure S30: HRMS (ESI) $[M+Na]^+$ spectrum of molecule **4**.

Figure S31: ^{1}H NMR spectrum of molecule **5**, acquired in 400 MHz spectrometer in the solvent $CDCl_3$ at 298K.

Figure S32: HRMS (ESI) $[M+Na]^+$ spectrum of molecule **5**.

Figure S33: ^{13}C NMR spectrum of molecule **6**, acquired in 800 MHz spectrometer in the solvent $CDCl_3$ at 298K.

Figure S34: 2D ^{1}H-^{13}C HSQC NMR spectrum of molecule **6**, acquired in 800 MHz spectrometer in the solvent $CDCl_3$ at 298K.

Figure S35: HRMS (ESI) $[M+Na]^+$ spectrum of molecule **6**.

Figure S36: ^{1}H NMR spectrum of molecule **7**, acquired in 400 MHz spectrometer in the solvent $CDCl_3$ at 298K.

Figure S37: ^{13}C NMR spectrum of molecule **7**, acquired in 800 MHz spectrometer in the solvent $CDCl_3$ at 298K.

Figure S38: 2D ^{1}H-^{13}C HSQC NMR spectrum of molecule **7**, acquired in 800 MHz spectrometer in the solvent $CDCl_3$ at 298K.

Figure S39: HRMS (ESI) $[M+Na]^+$ spectrum of molecule **7**.

Figure S40: The DFT optimized spatial structure ((a) and (b) are two different projections) of molecule **1**.

Figure S41: The DFT optimized spatial structure ((a) and (b) are two different projections) of molecule **3**.

Figure S42: The DFT optimized spatial structure ((a) and (b) are two different projections) of molecule **4**.

Figure S43: The DFT optimized spatial structure ((a) and (b) are two different projections) of molecule **5**.

Figure S44: The DFT optimized spatial structure ((a) and (b) are two different projections) of molecule **6**.

Figure S45: The DFT optimized spatial structure ((a) and (b) are two different projections) of molecule **7**.

Table S1: The amide temperature coefficients of molecules, **1-3.**

Table S2: Crystal data and structure refinement for molecule **2**.

Table S3: The comparision of theoretical chemical shift of NH^1 (δ_{NH1}) obtained from the DFT optimized spatial structure and the experimental values for molecules **1** to **7**.

S46: Experimental

S48: References

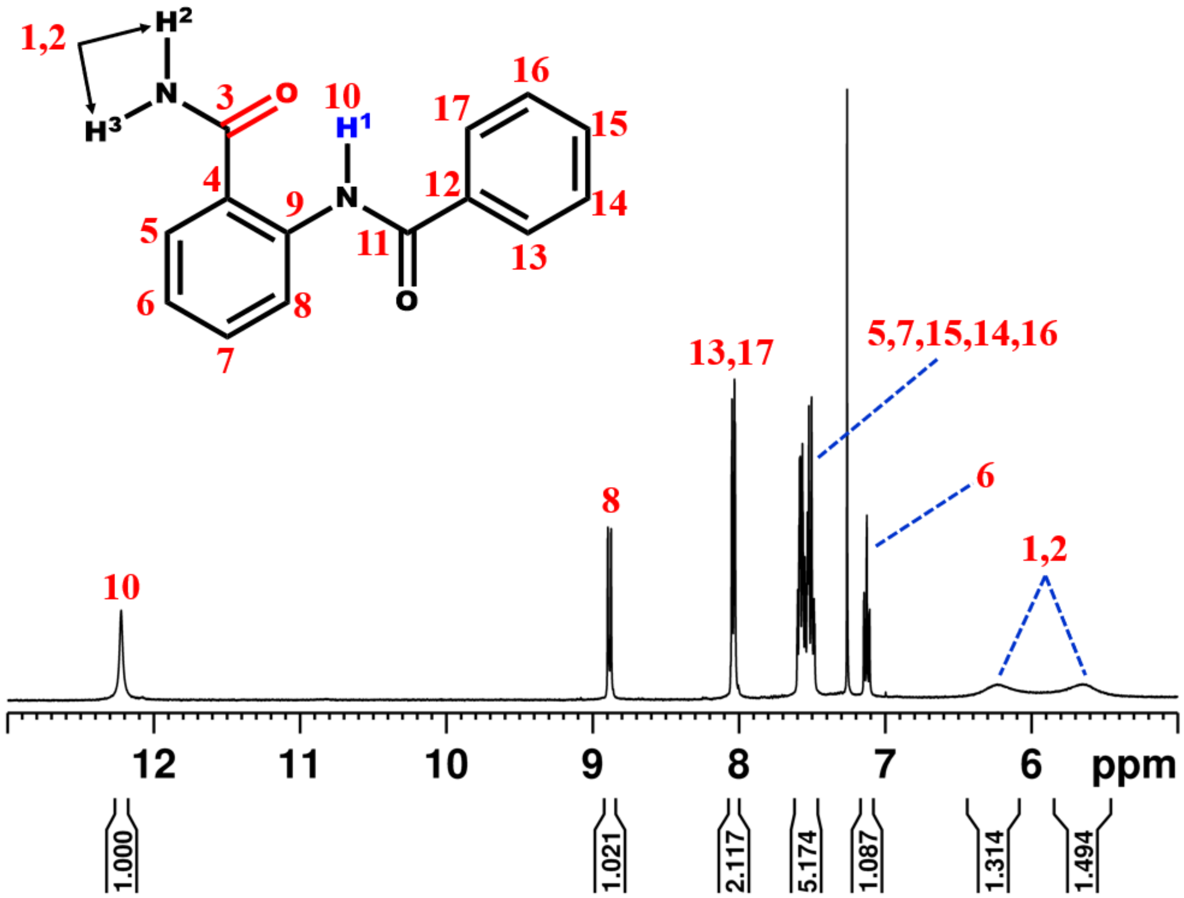

Figure S1: ^{1}H NMR spectrum of molecule **1**, acquired in 400 MHz spectrometer in the solvent $CDCl_3$ at 298K.

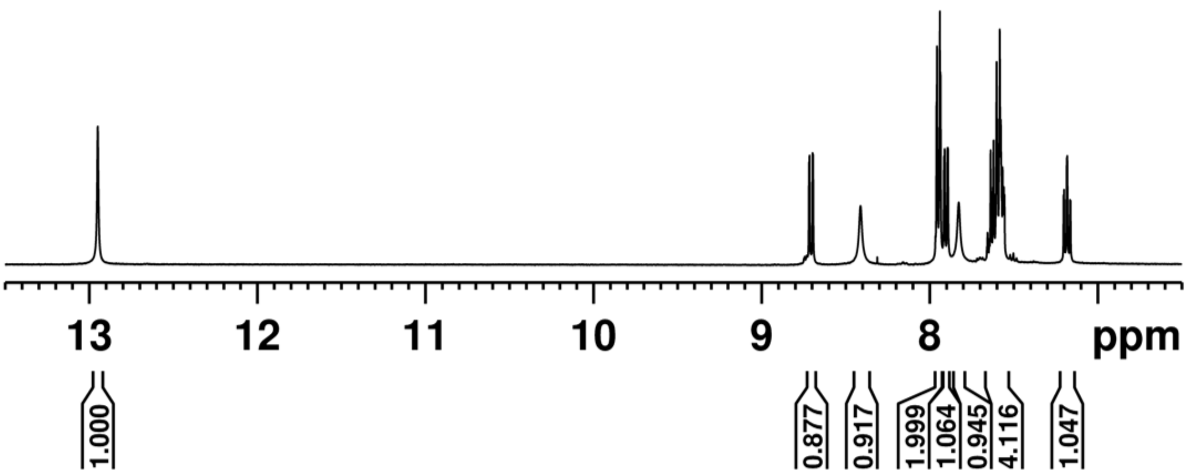

Figure S2: ^{1}H NMR spectrum of molecule **1**, acquired in 400 MHz spectrometer in the solvent DMSO at 298K

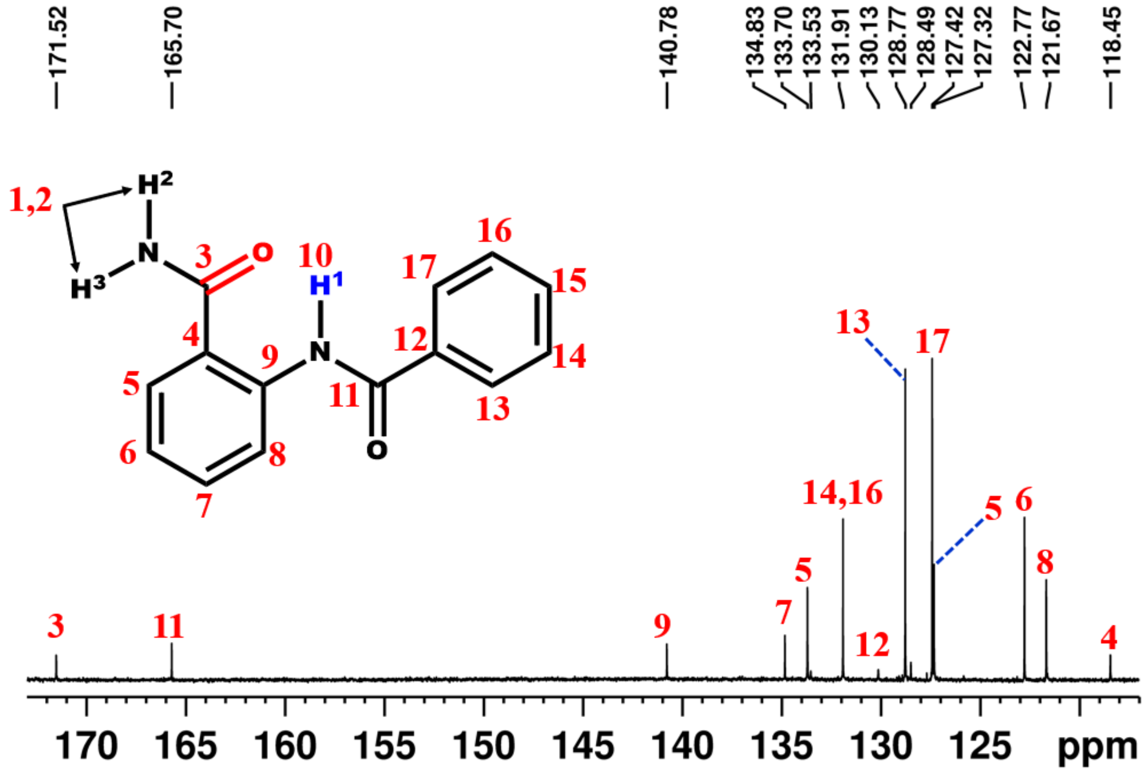

Figure S3: ^{13}C NMR spectrum of molecule **1**, acquired in 800 MHz spectrometer in the solvent $CDCl_3$ at 298K.

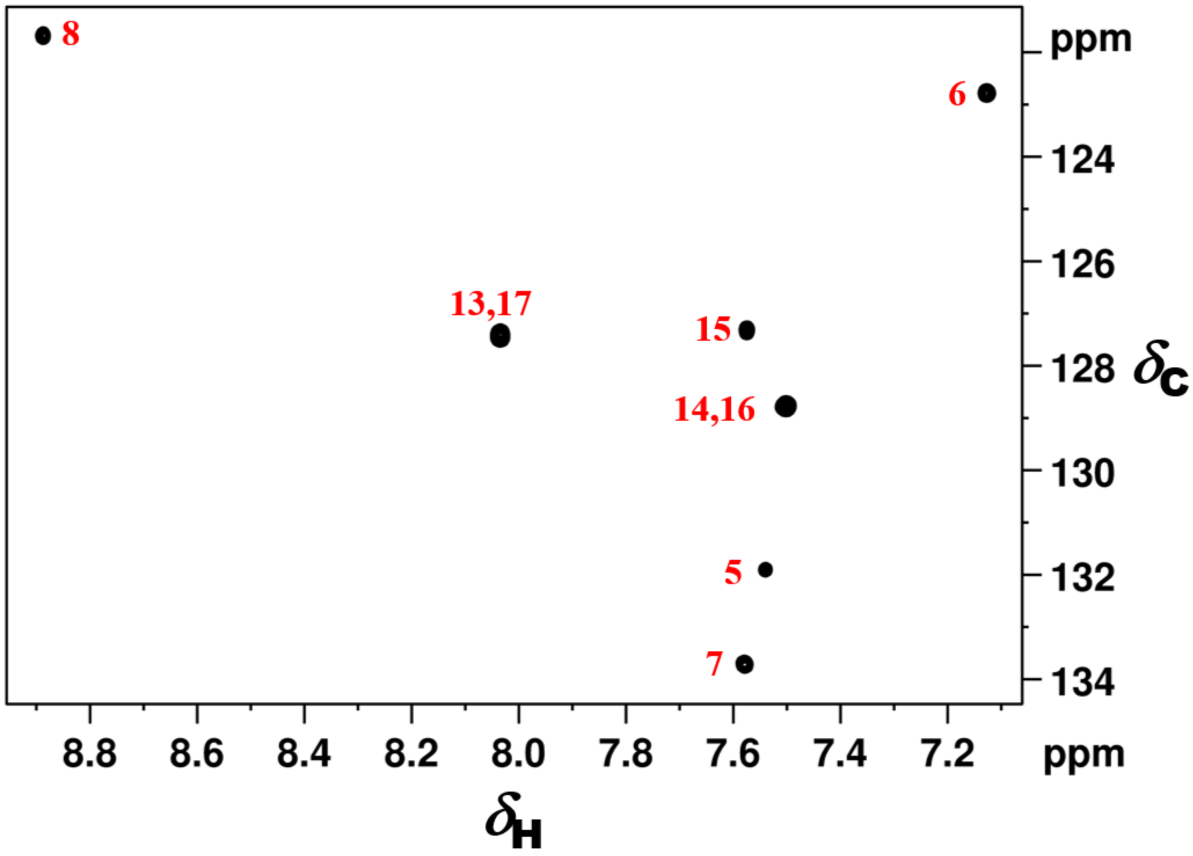

Figure S4: 2D ^{1}H-^{13}C HSQC NMR spectrum of molecule **1**, acquired in 800 MHz spectrometer in the solvent $CDCl_3$ at 298K.

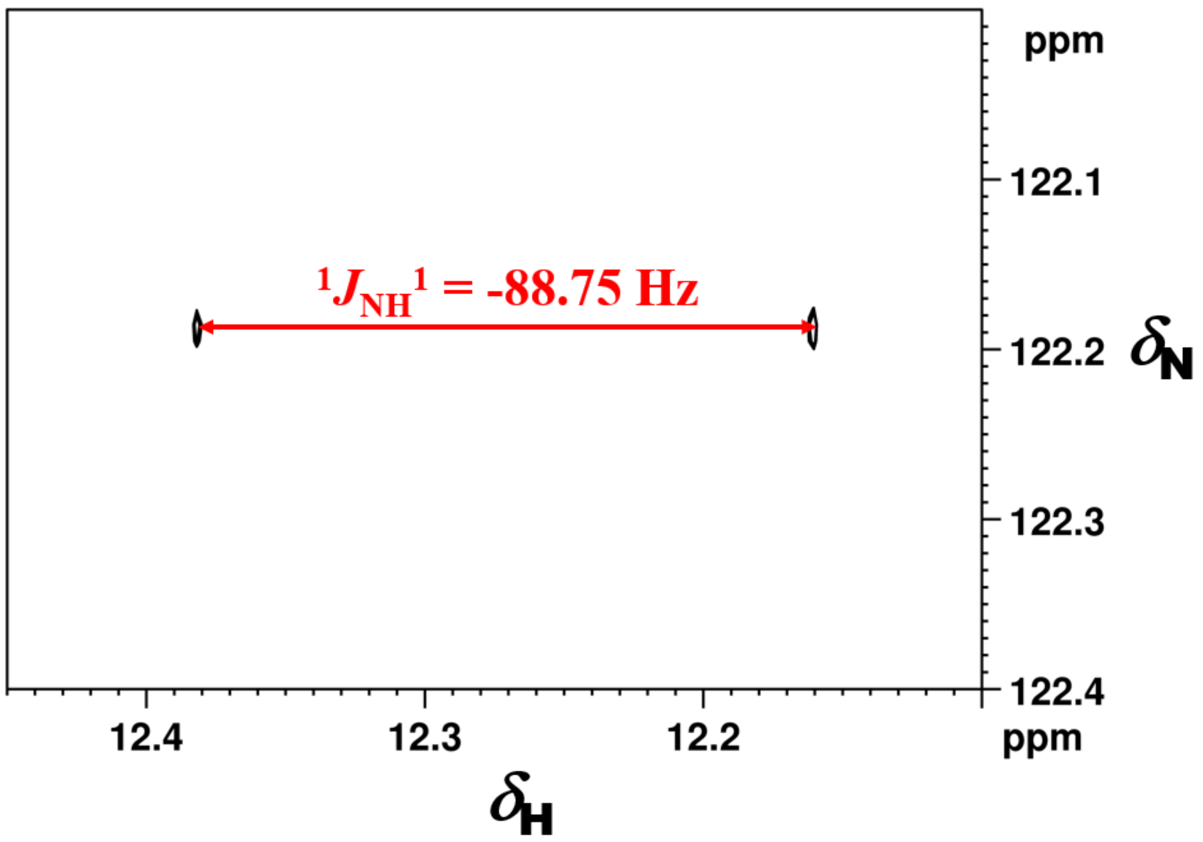

Figure S5: 2D ^1H-^{15}N coupled HSQC NMR spectrum of molecule **1** of NH^1 region, acquired in 400 MHz spectrometer in the solvent $CDCl_3$ at 298K.

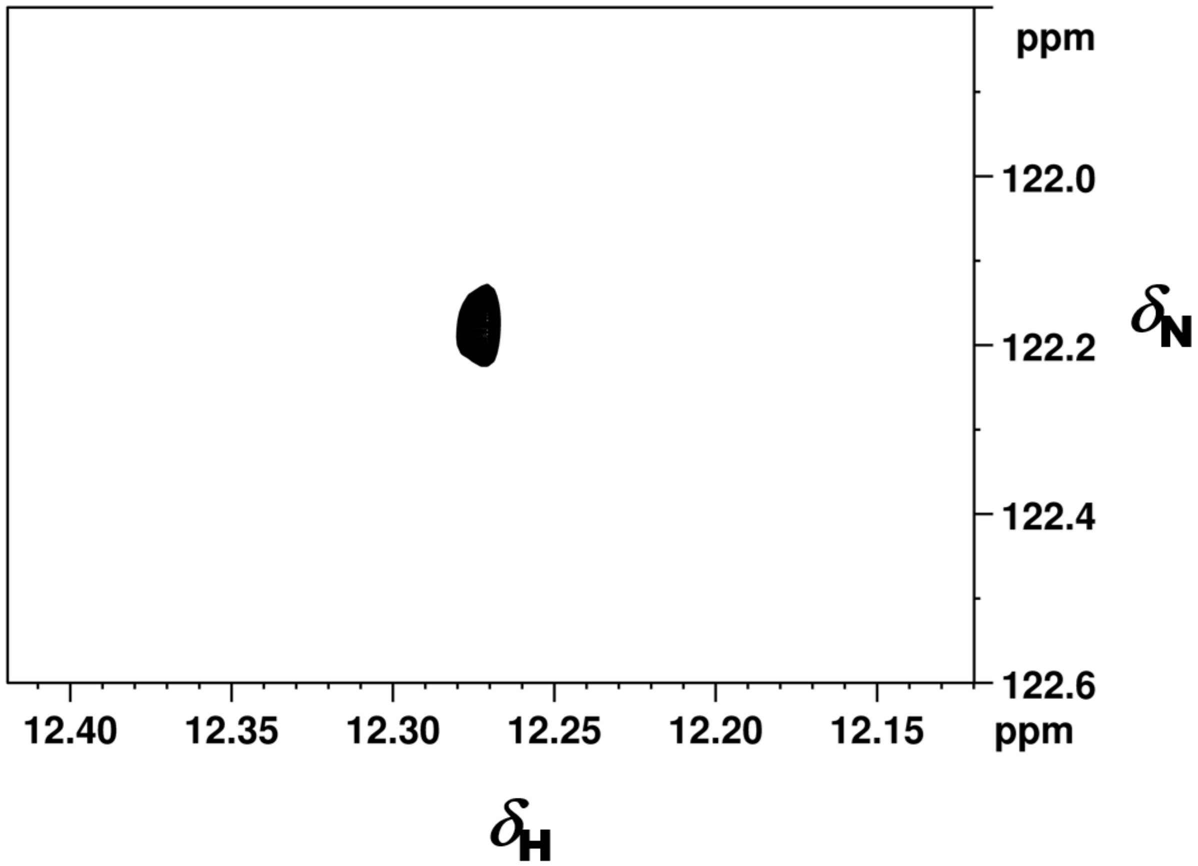

Figure S6: 2D ^{1}H-^{15}N decoupled HSQC NMR spectrum of molecule **1** of NH^{1} region, acquired in 400 MHz spectrometer in the solvent $CDCl_3$ at 298K.

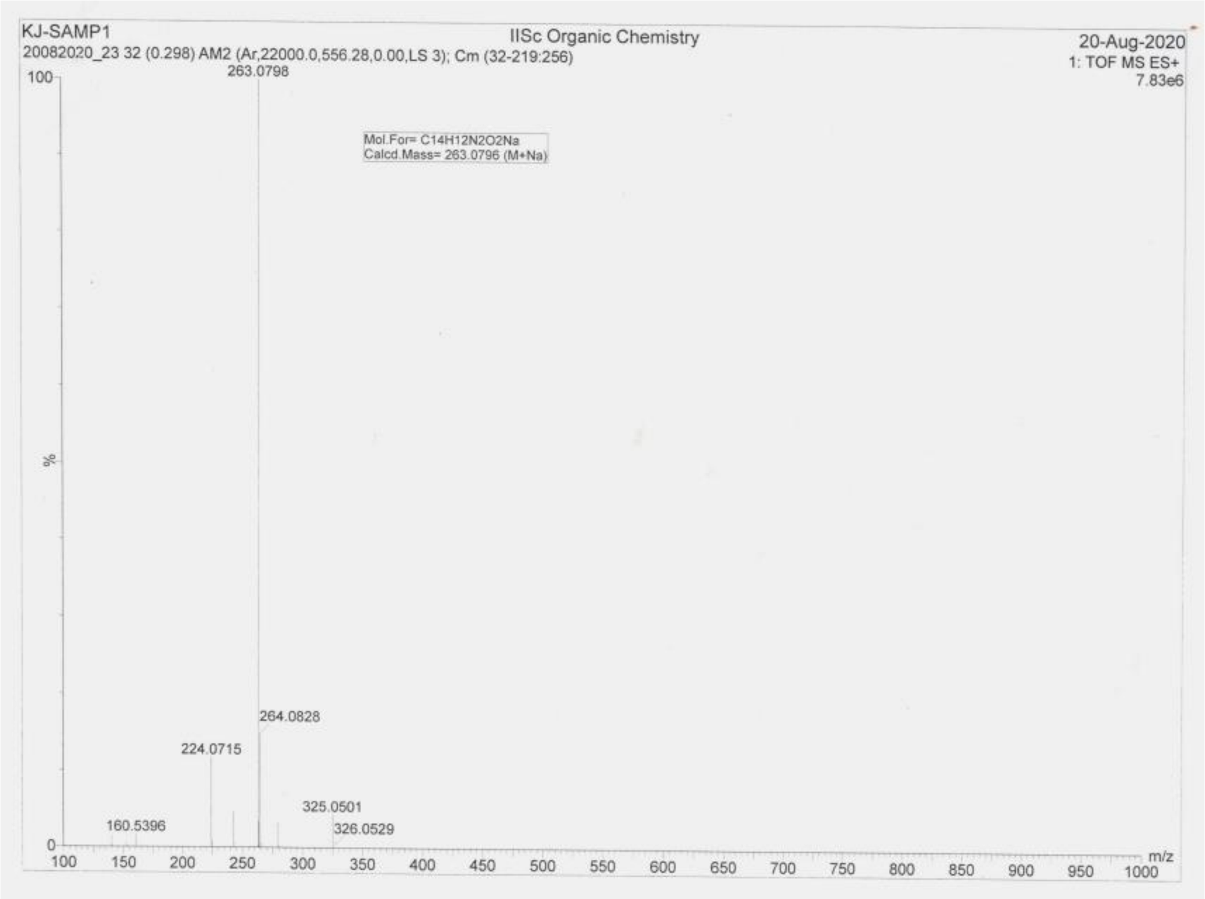

Figure S7: HRMS (ESI) $[M+Na]^+$ spectrum of molecule **1**.

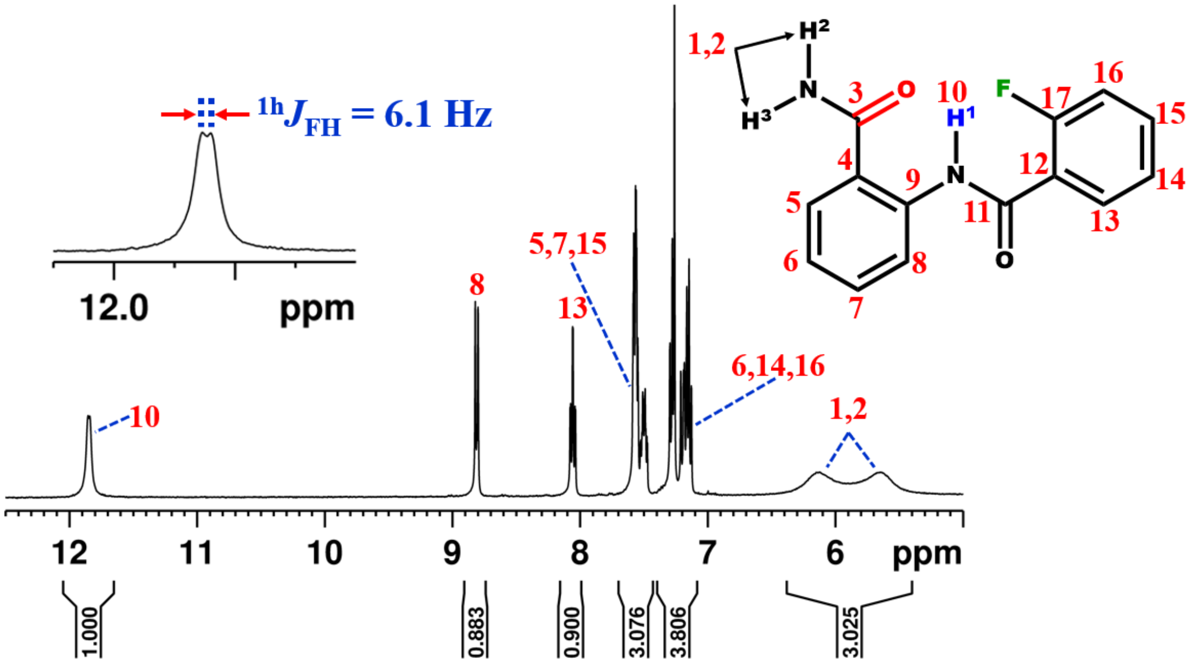

Figure S8: ^{1}H NMR spectrum of molecule **2**, acquired in 400 MHz spectrometer in the solvent $CDCl_3$ at 298K.

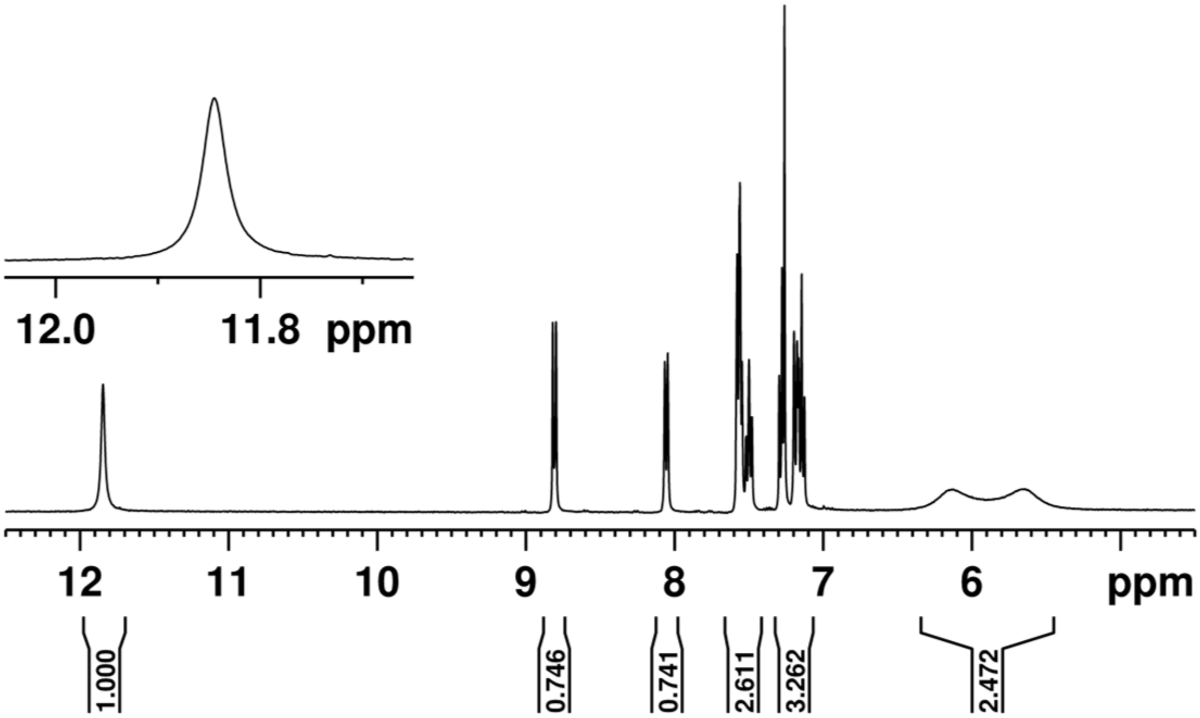

Figure S9: $^{1}H\{^{19}F\}$ NMR spectrum of molecule **2**, acquired in 400 MHz spectrometer in the solvent $CDCl_3$ at 298K.

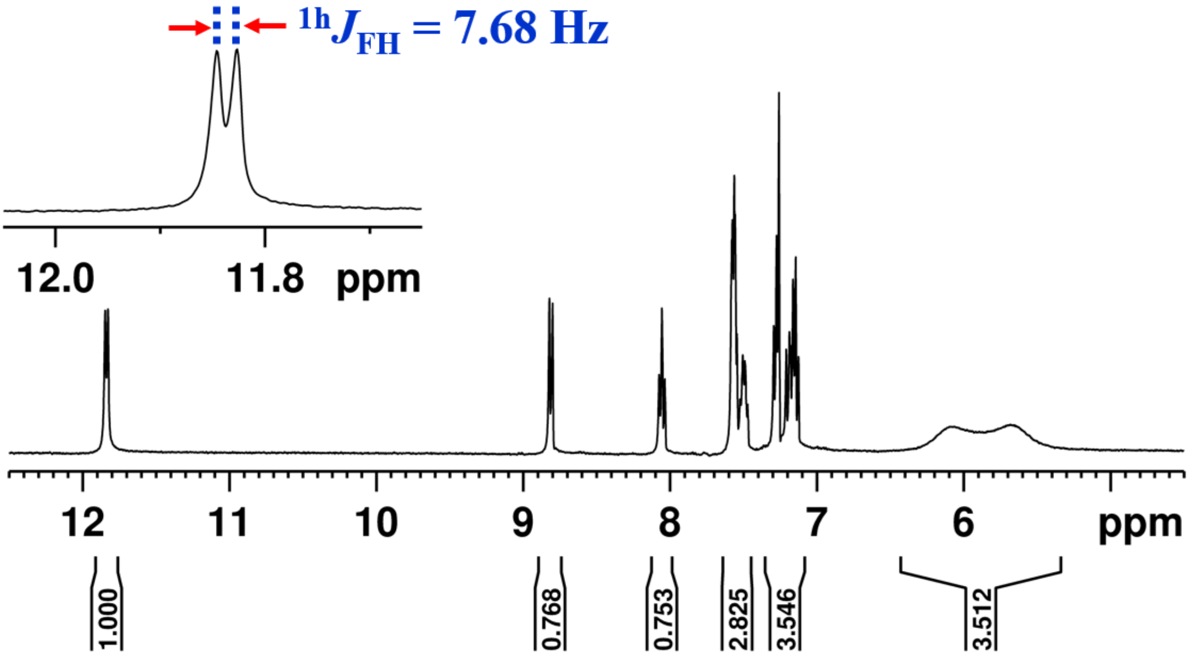

Figure S10: $^1H\{^{14}N\}$ NMR spectrum of molecule **2**, acquired in 400 MHz spectrometer in the solvent $CDCl_3$ at 298K.

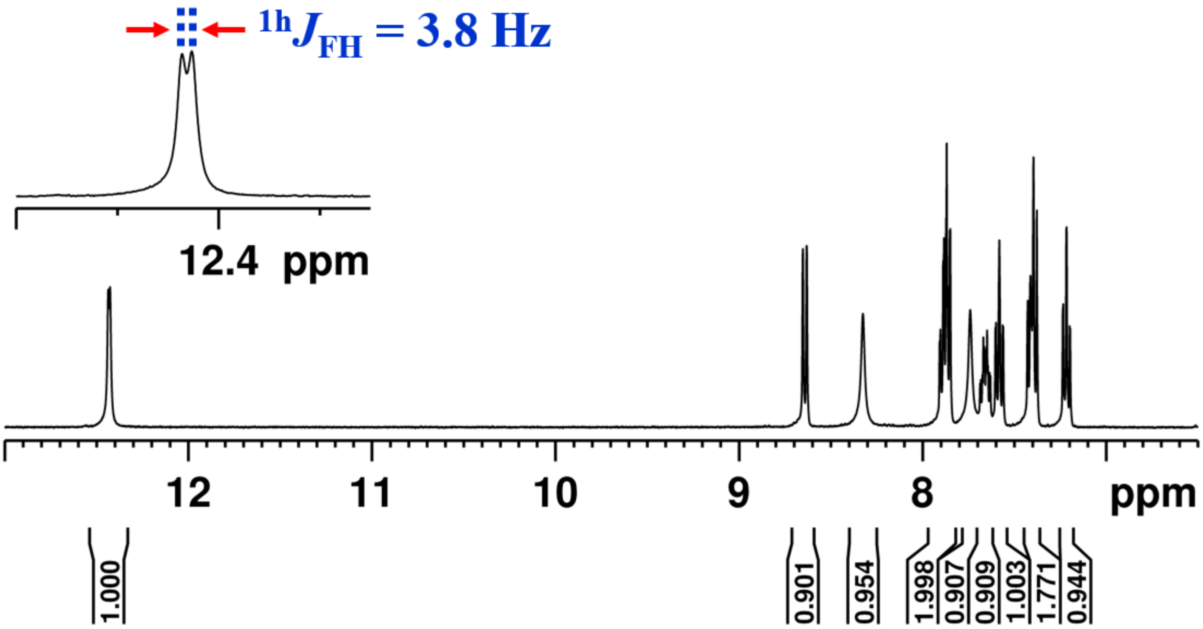

Figure S11: ^1H NMR spectrum of molecule **2**, acquired in 400 MHz spectrometer in the solvent DMSO at 298K.

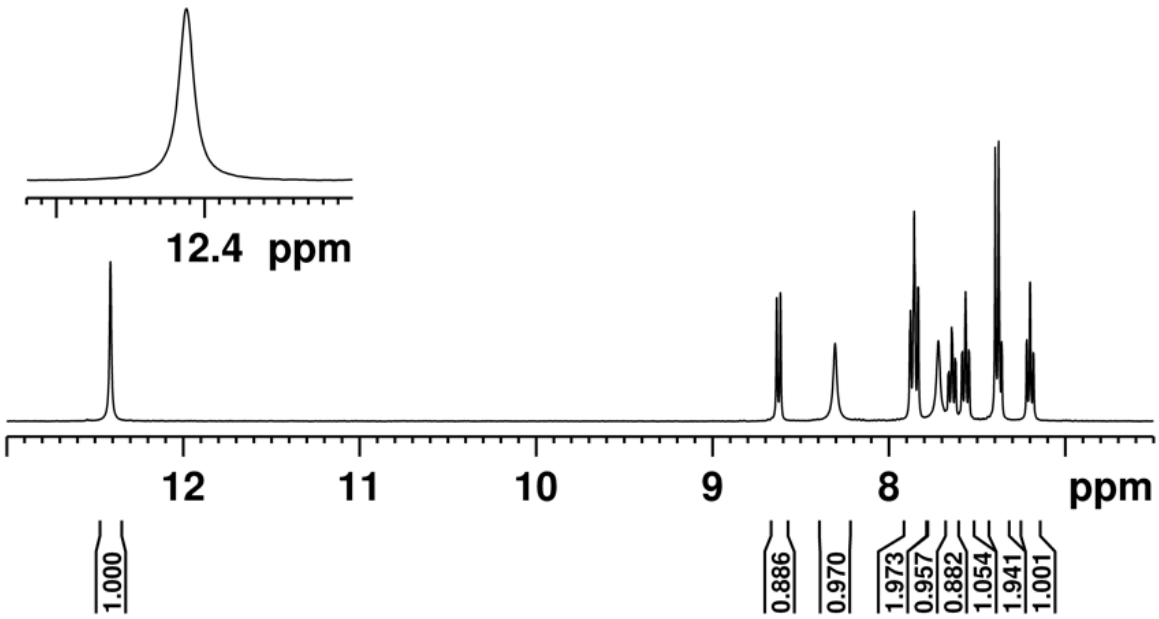

Figure S12: $^{1}H\{^{19}F\}$ NMR spectrum of molecule **2**, acquired in 400 MHz spectrometer in the solvent DMSO at 298K.

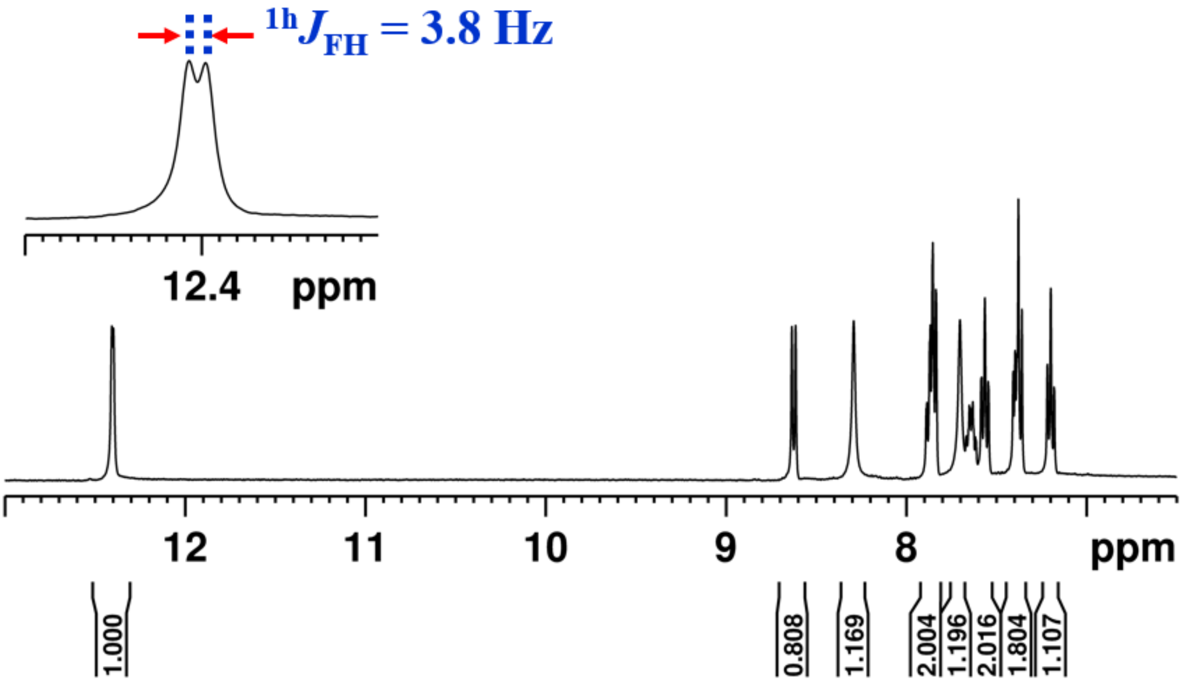

Figure S13: $^{1}H\{^{14}N\}$ NMR spectrum of molecule **2**, acquired in 400 MHz spectrometer in the solvent DMSO at 298K.

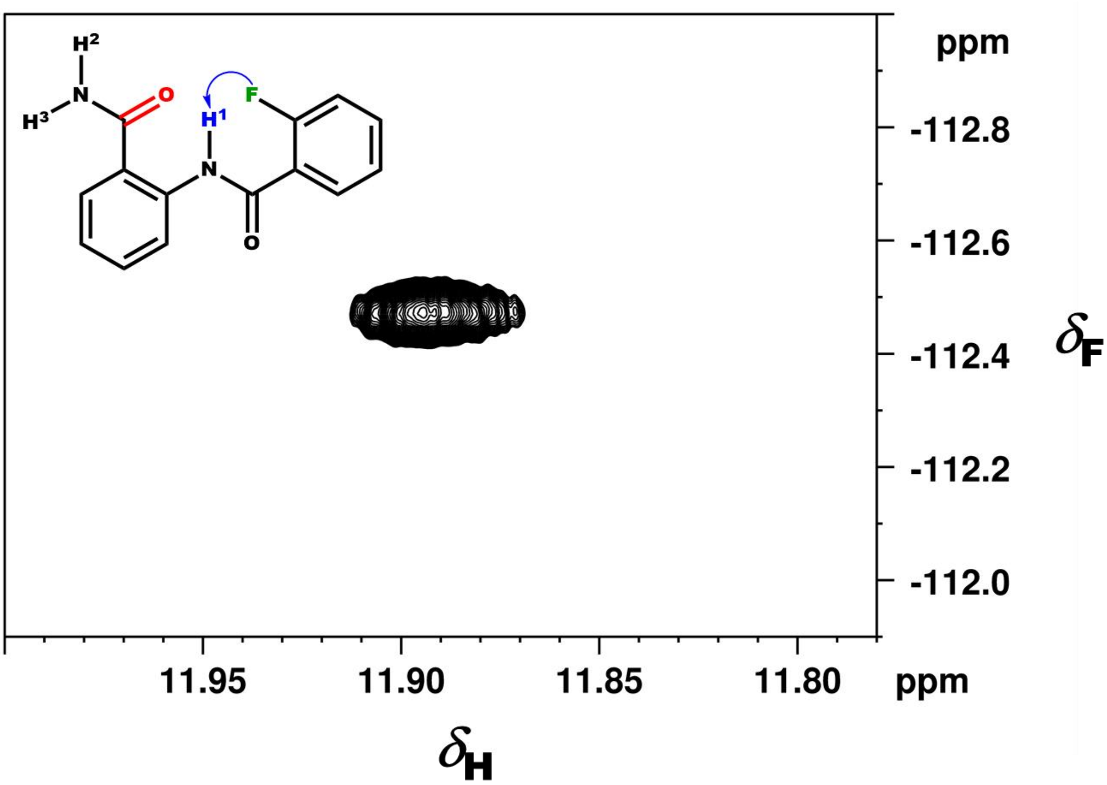

Figure S14: 400 MHz two dimensional ^{19}F-^{1}H HOESY spectrum of fluorine substituted molecule (molecule **2)** of NH^1 region in the solvent $CDCl_3$ at 298 K.

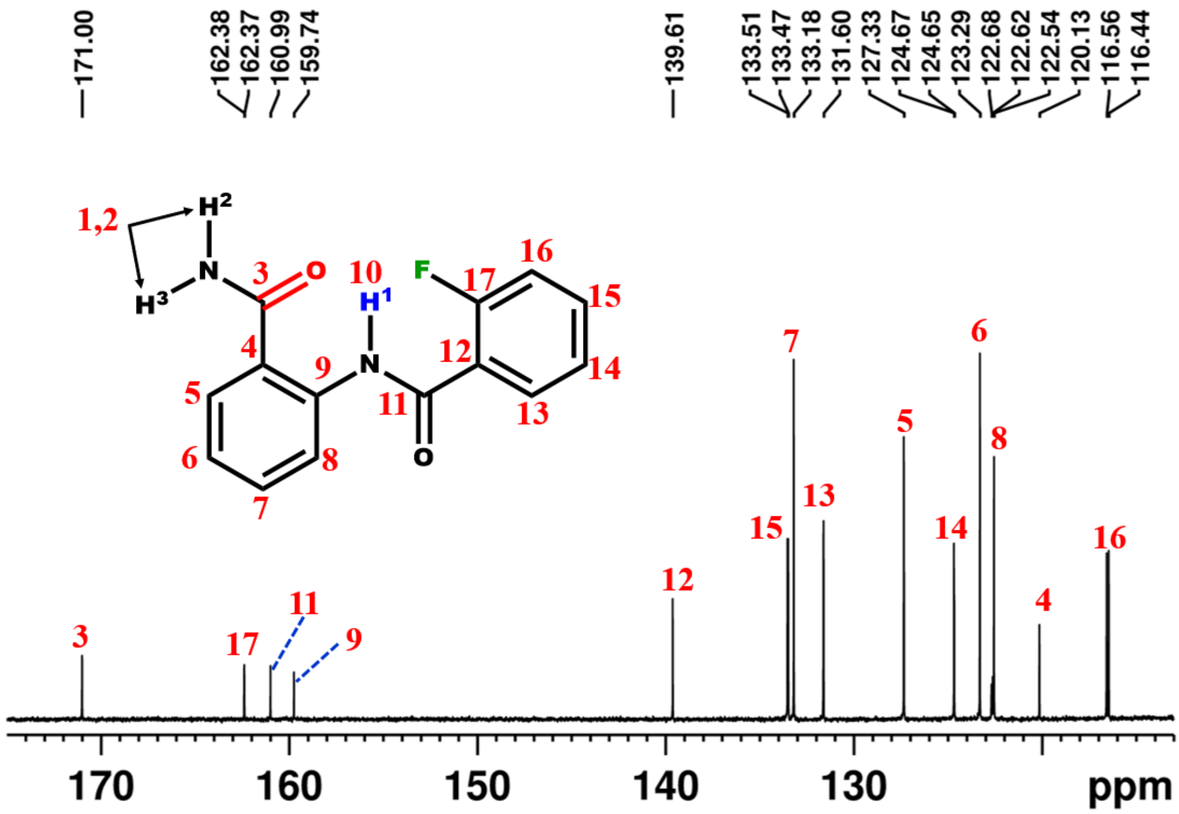

Figure S15: ^{13}C NMR spectrum of molecule **2**, acquired in 800 MHz spectrometer in the solvent $CDCl_3$ at 298K.

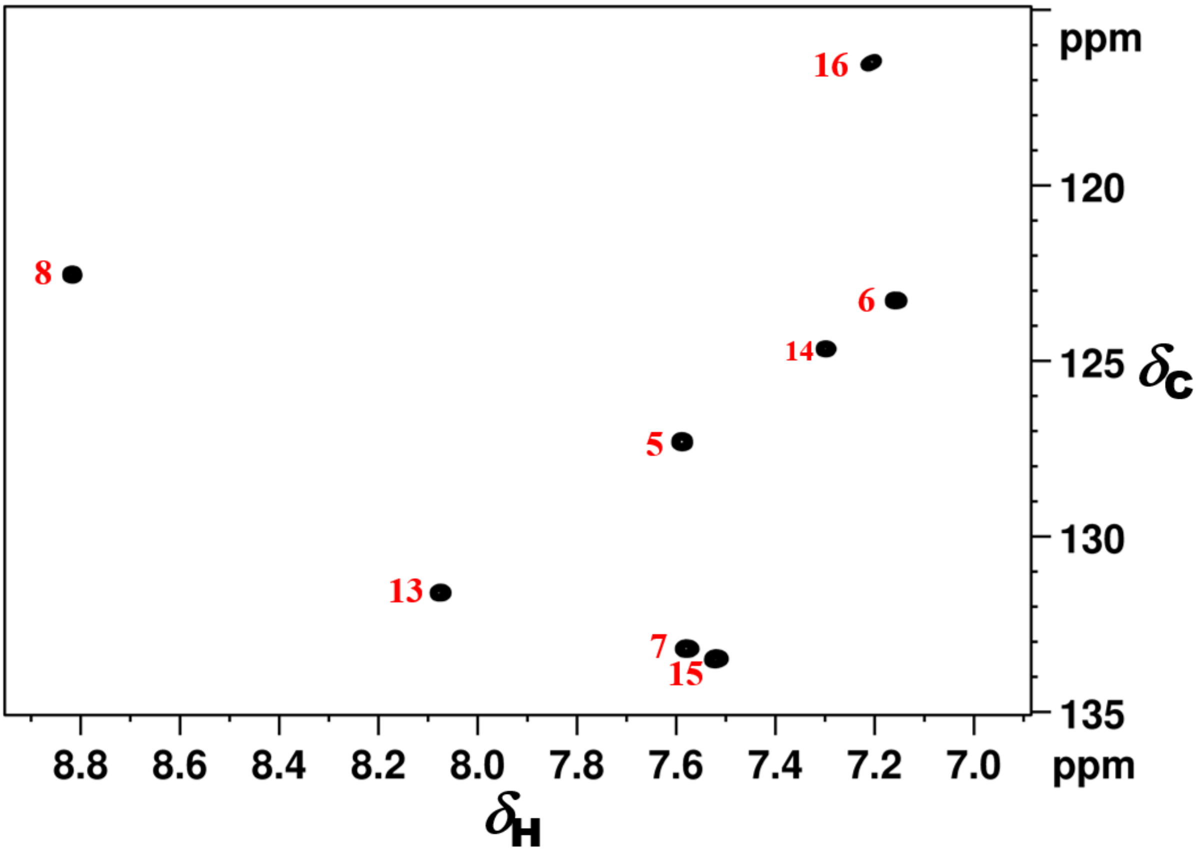

Figure S16: 2D ^{1}H-^{13}C HSQC NMR spectrum of molecule **2**, acquired in 800 MHz spectrometer in the solvent $CDCl_3$ at 298K.

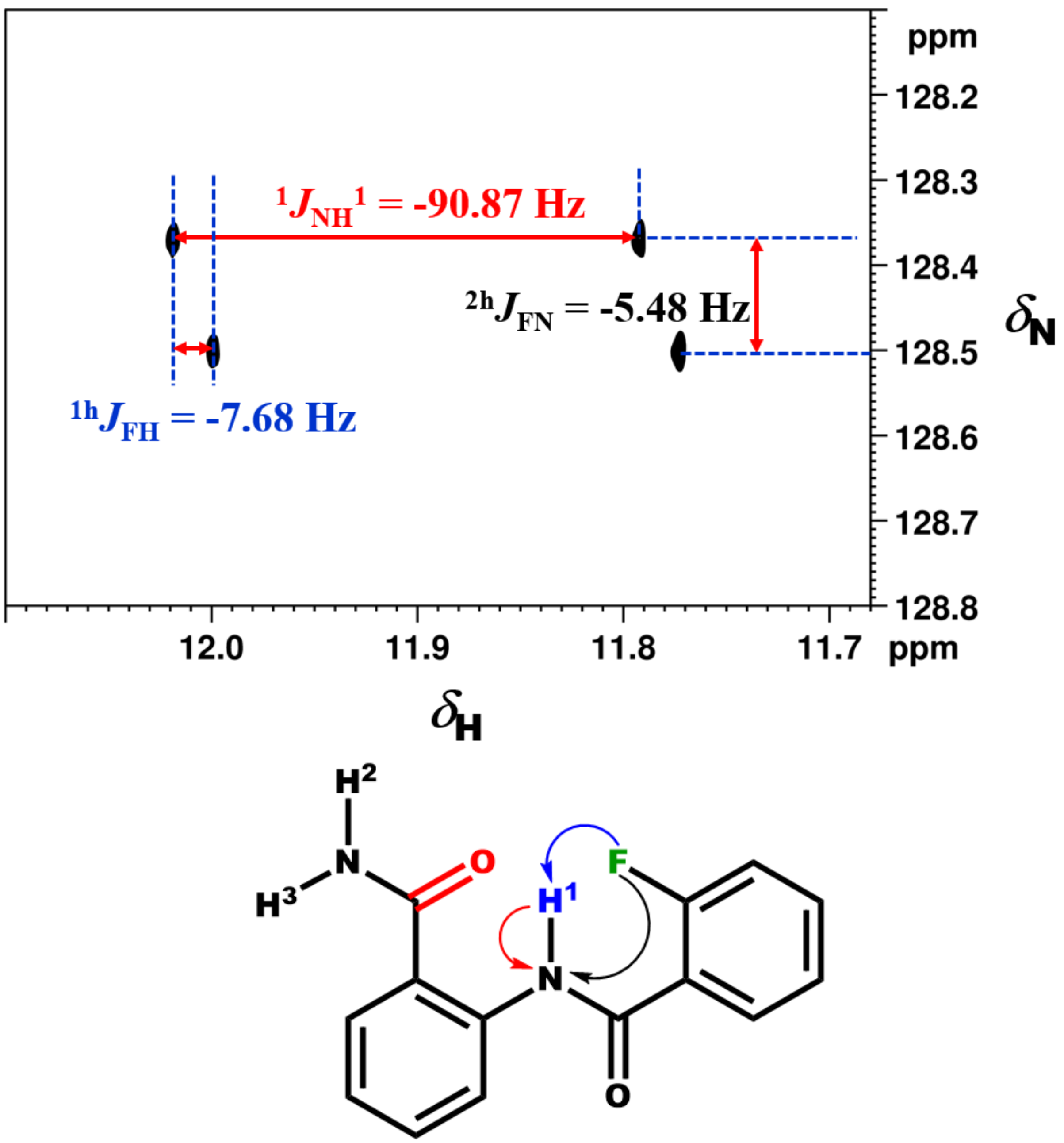

Figure S17: 2D ^1H-^{15}N coupled HSQC NMR spectrum of molecule **2** of NH^1 region, acquired in 400 MHz spectrometer in the solvent $CDCl_3$ at 298K. The chemical structure of the molecule shows the couplings $^1J_{NH}$, $^{1h}J_{FH}$ and $^{2h}J_{FN}$ and are identified by arrows red, blue and black respectively.

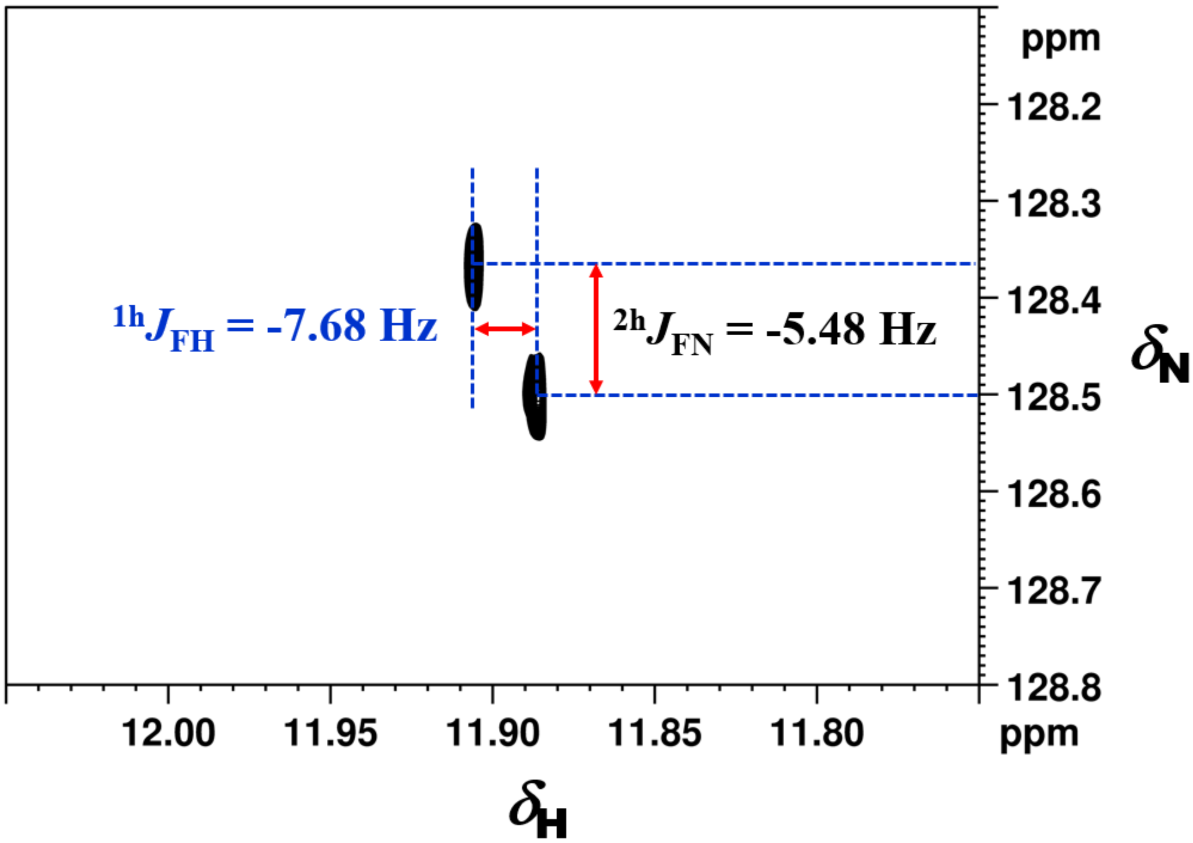

Figure S18: 2D ^1H-^{15}N decoupled HSQC NMR spectrum of molecule **2** of NH^1 region, acquired in 400 MHz spectrometer in the solvent $CDCl_3$ at 298K.

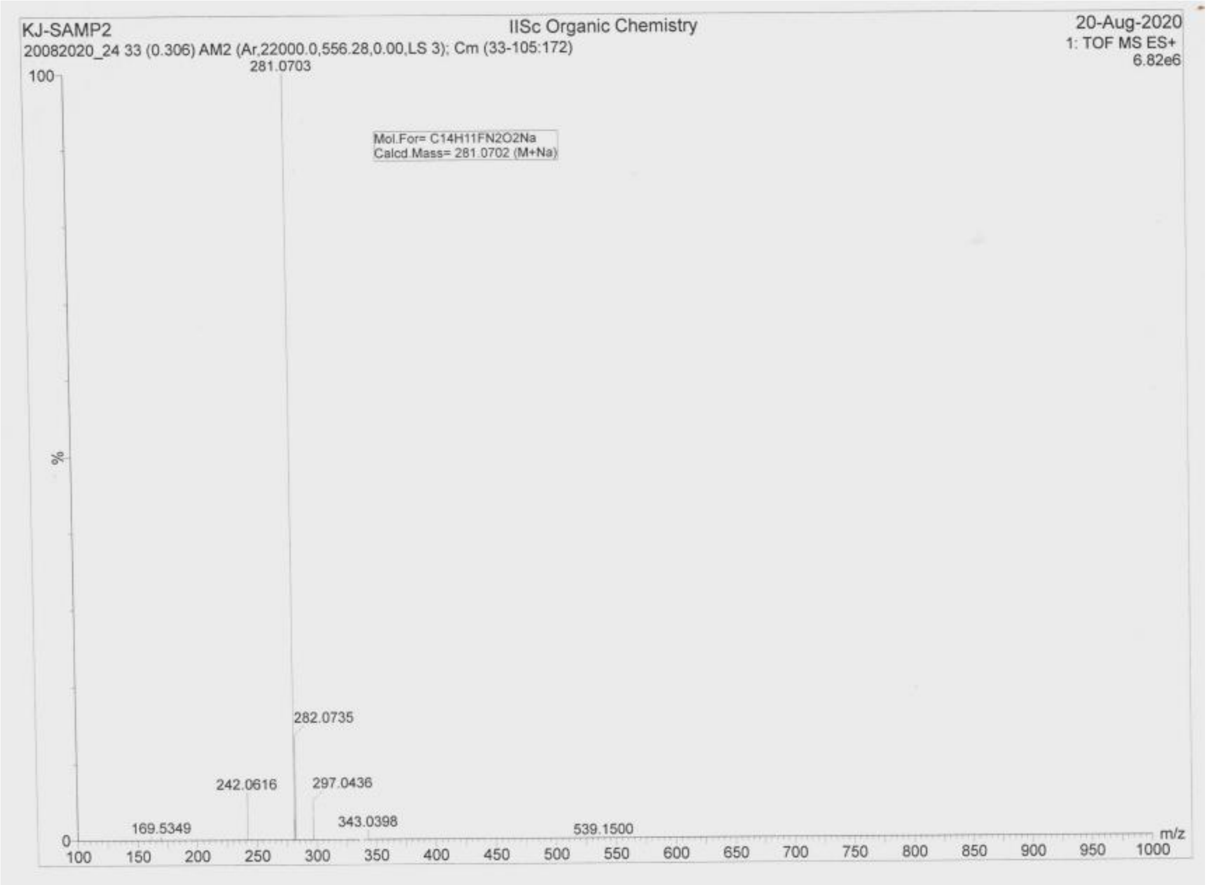

Figure S19: HRMS (ESI) $[M+Na]^+$ spectrum of molecule **2**.

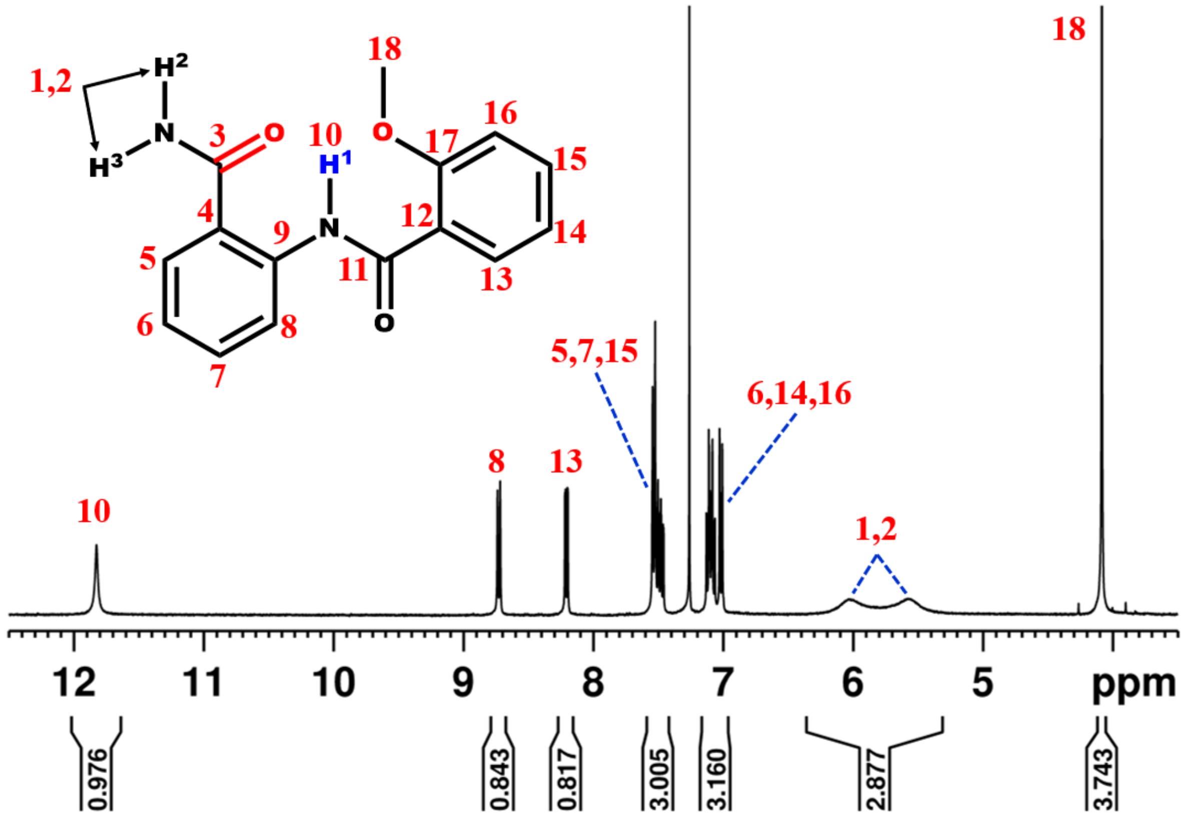

Figure S20: ^{1}H NMR spectrum of molecule **3**, acquired in 400 MHz spectrometer in the solvent $CDCl_3$ at 298K.

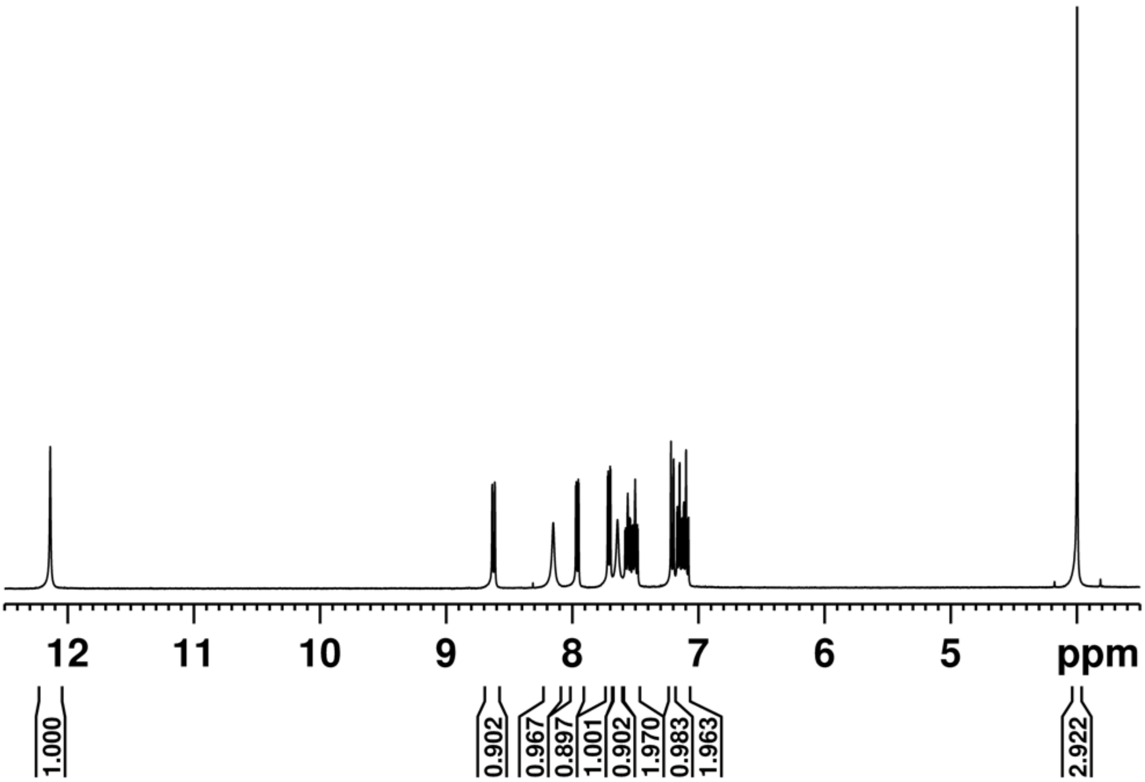

Figure S21: ^{1}H NMR spectrum of molecule **3**, acquired in 400 MHz spectrometer in the solvent DMSO at 298K.

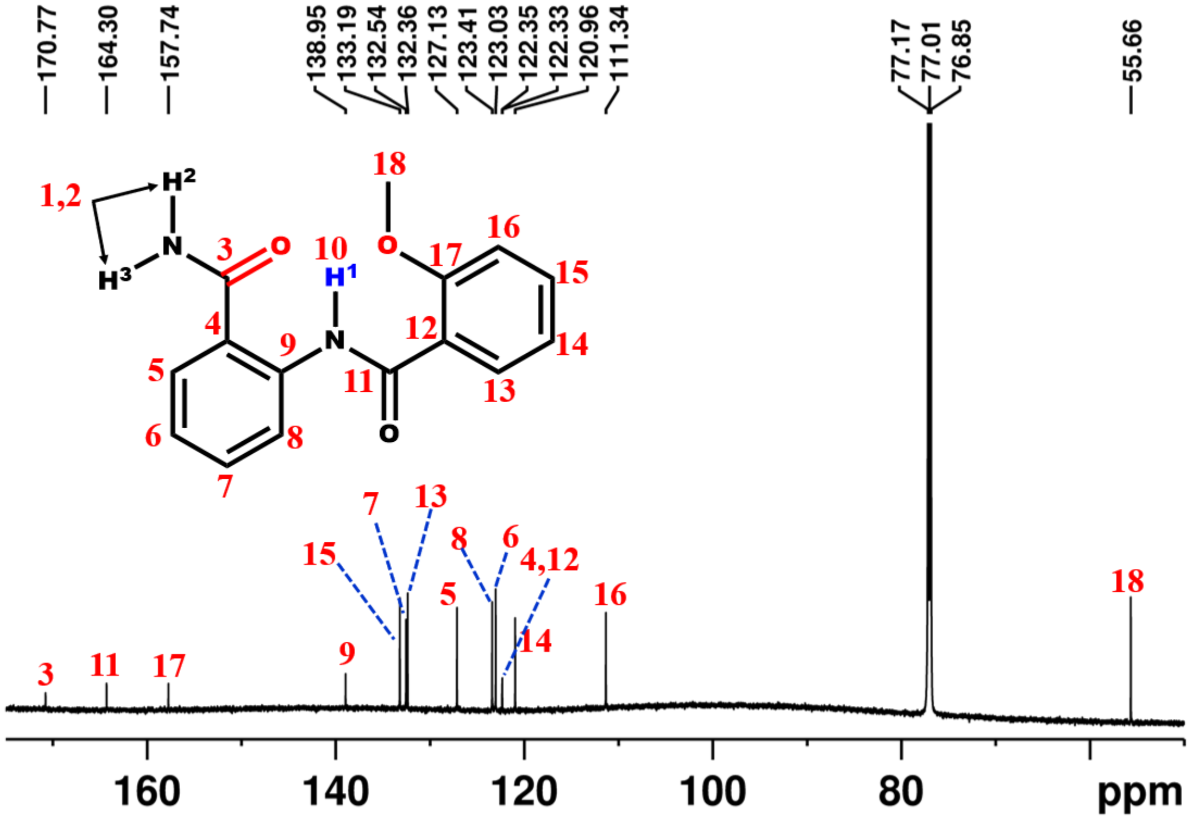

Figure S22: ^{13}C NMR spectrum of molecule **3**, acquired in 800 MHz spectrometer in the solvent $CDCl_3$ at 298K.

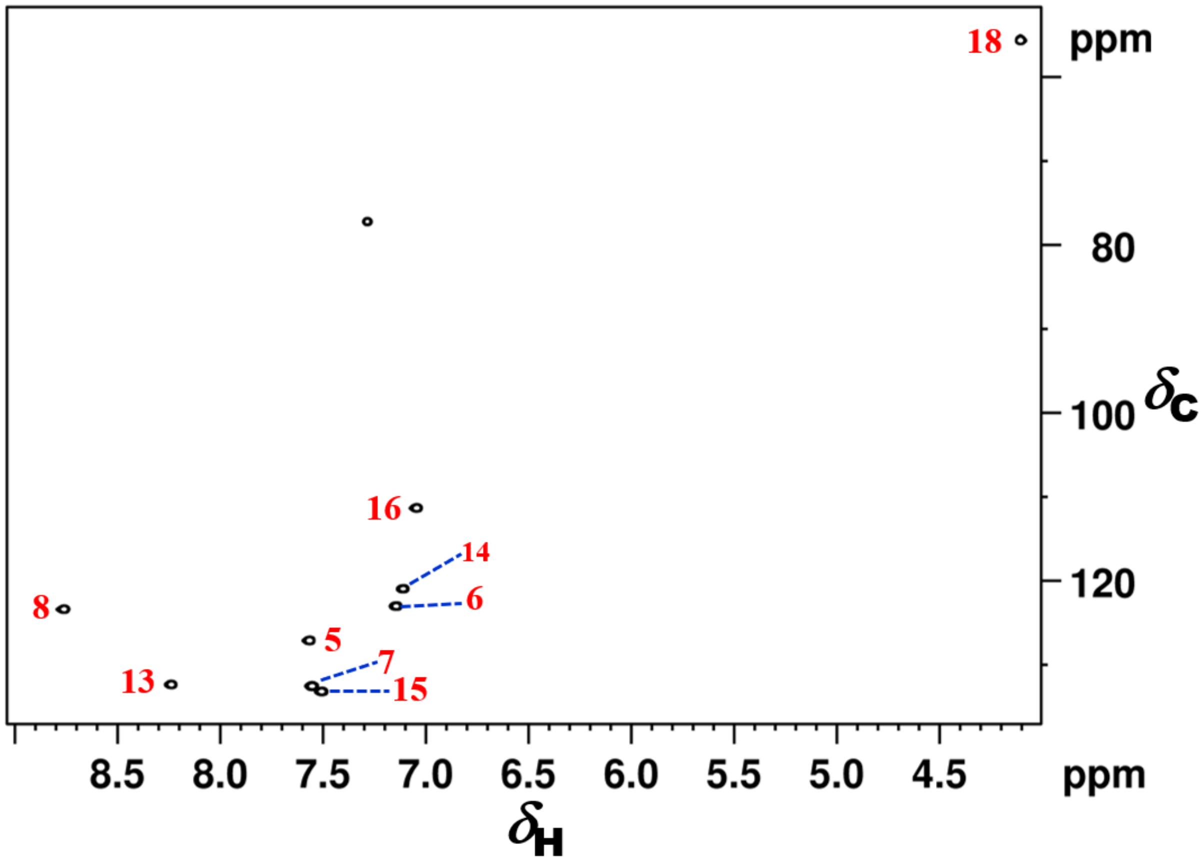

Figure S23: 2D ^{1}H-^{13}C HSQC NMR spectrum of molecule **3**, acquired in 800 MHz spectrometer in the solvent $CDCl_3$ at 298K.

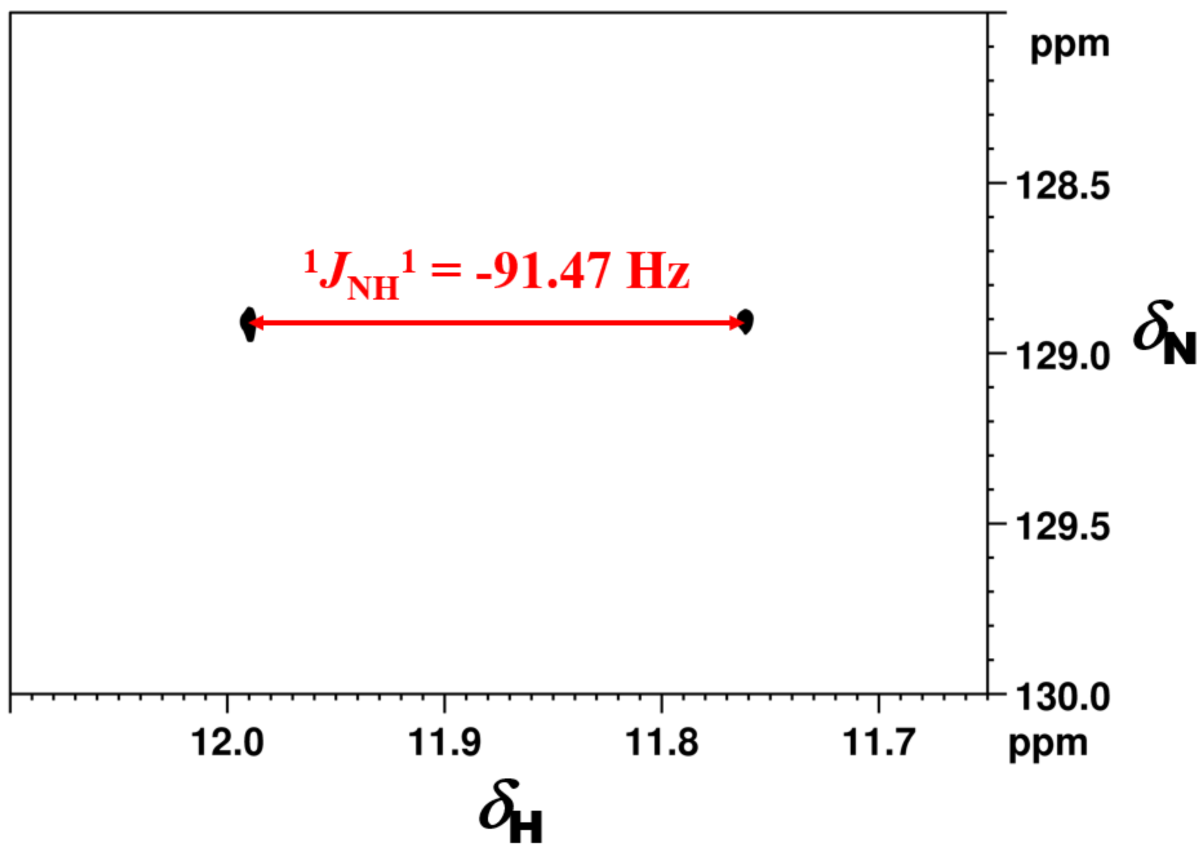

Figure S24: 2D ^1H-^{15}N coupled HSQC NMR spectrum of molecule **3** of NH^1 region, acquired in 400 MHz spectrometer in the solvent $CDCl_3$ at 298K.

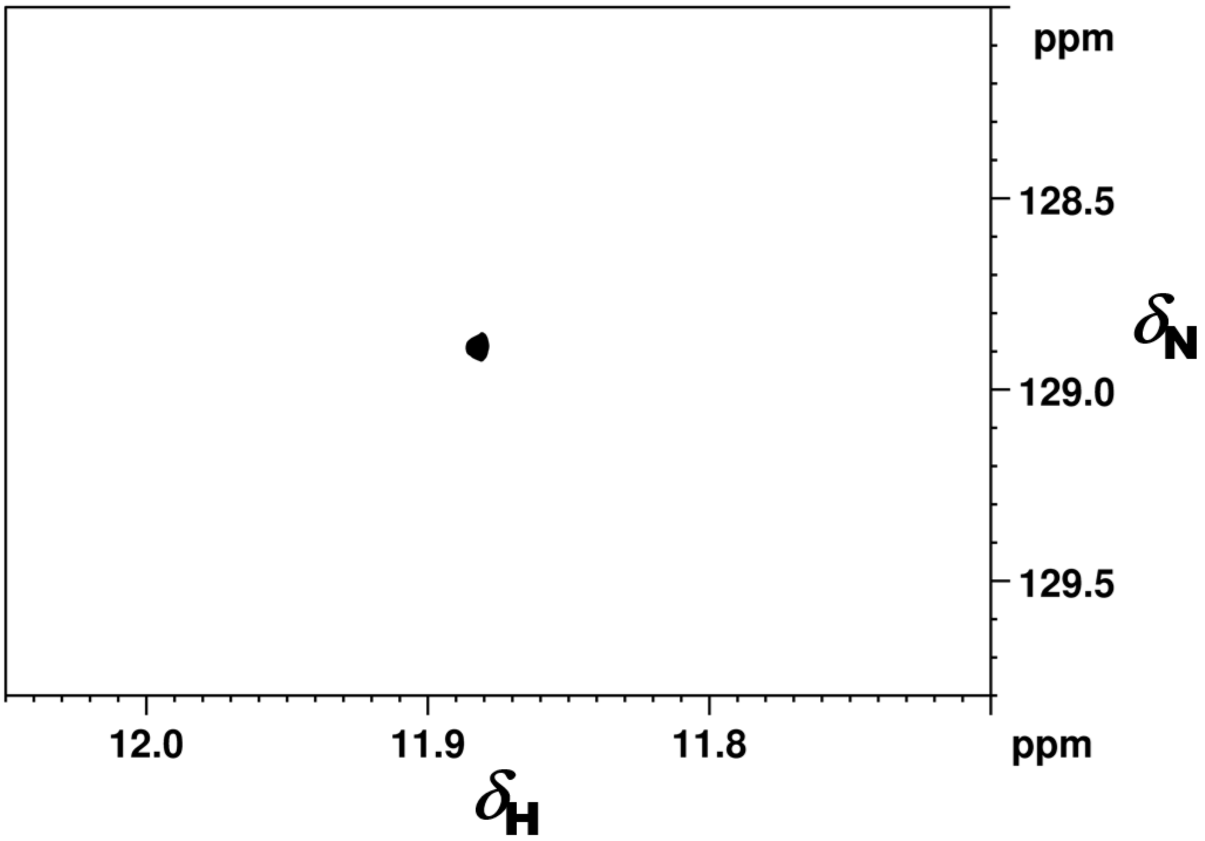

Figure S25: 2D ^1H-^{15}N decoupled HSQC NMR spectrum of molecule **3** of NH^1 region, acquired in 400 MHz spectrometer in the solvent $CDCl_3$ at 298K.

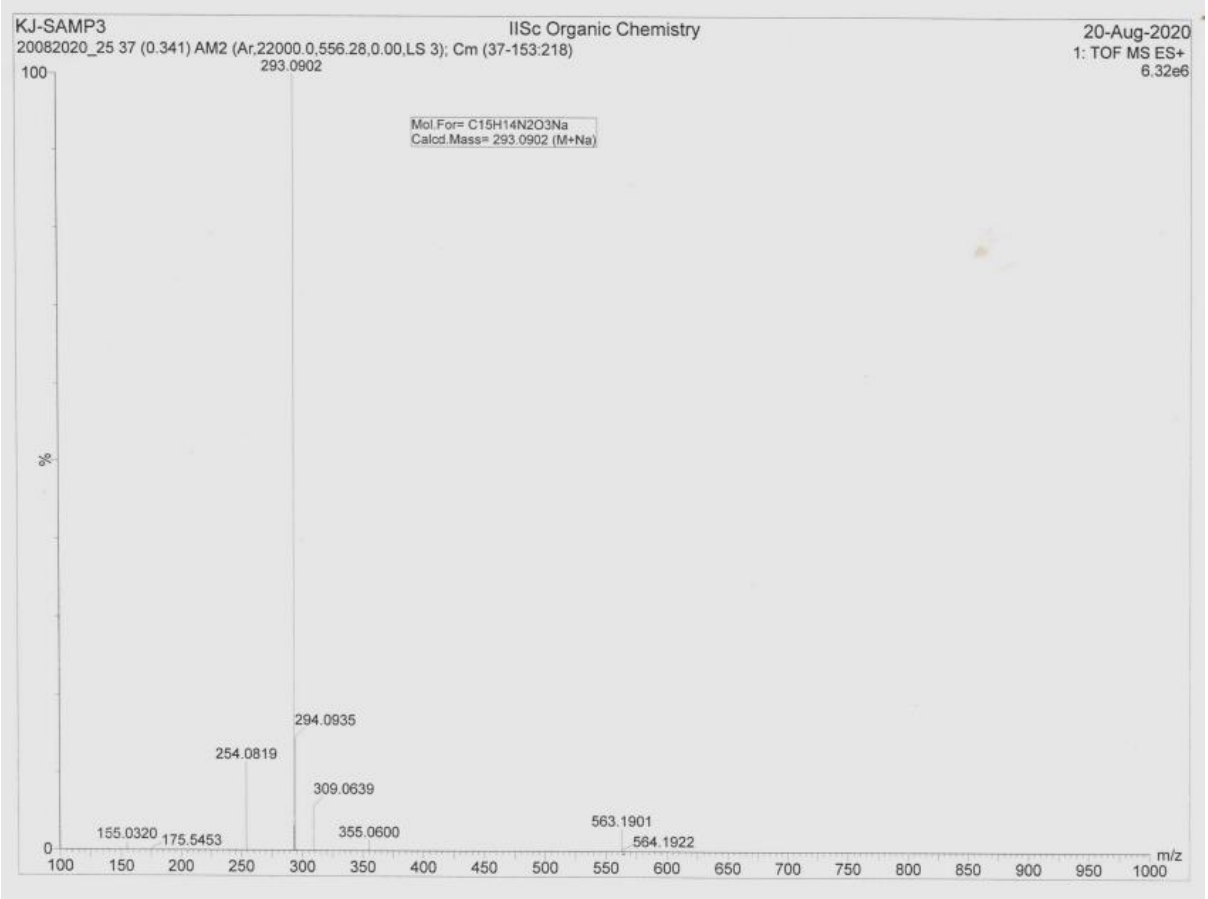

Figure S26: HRMS (ESI) $[M+Na]^+$ spectrum of molecule **3**.

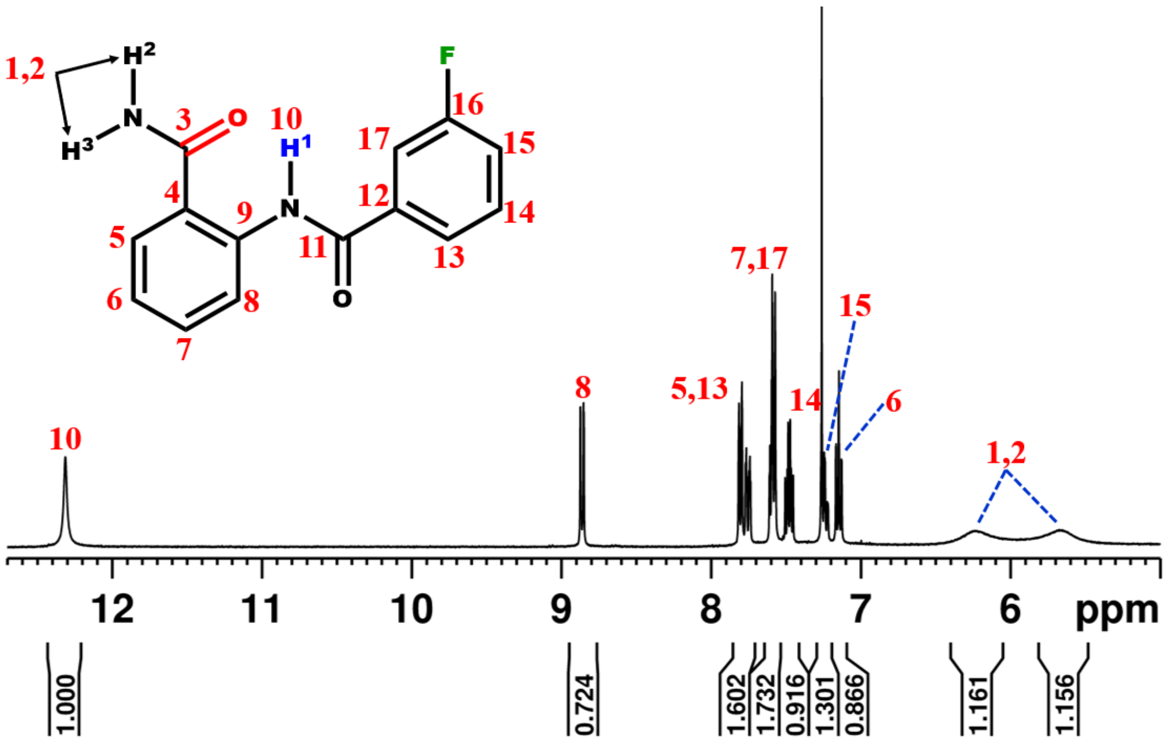

Figure S27: ^{1}H NMR spectrum of molecule **4**, acquired in 400 MHz spectrometer in the solvent $CDCl_3$ at 298K.

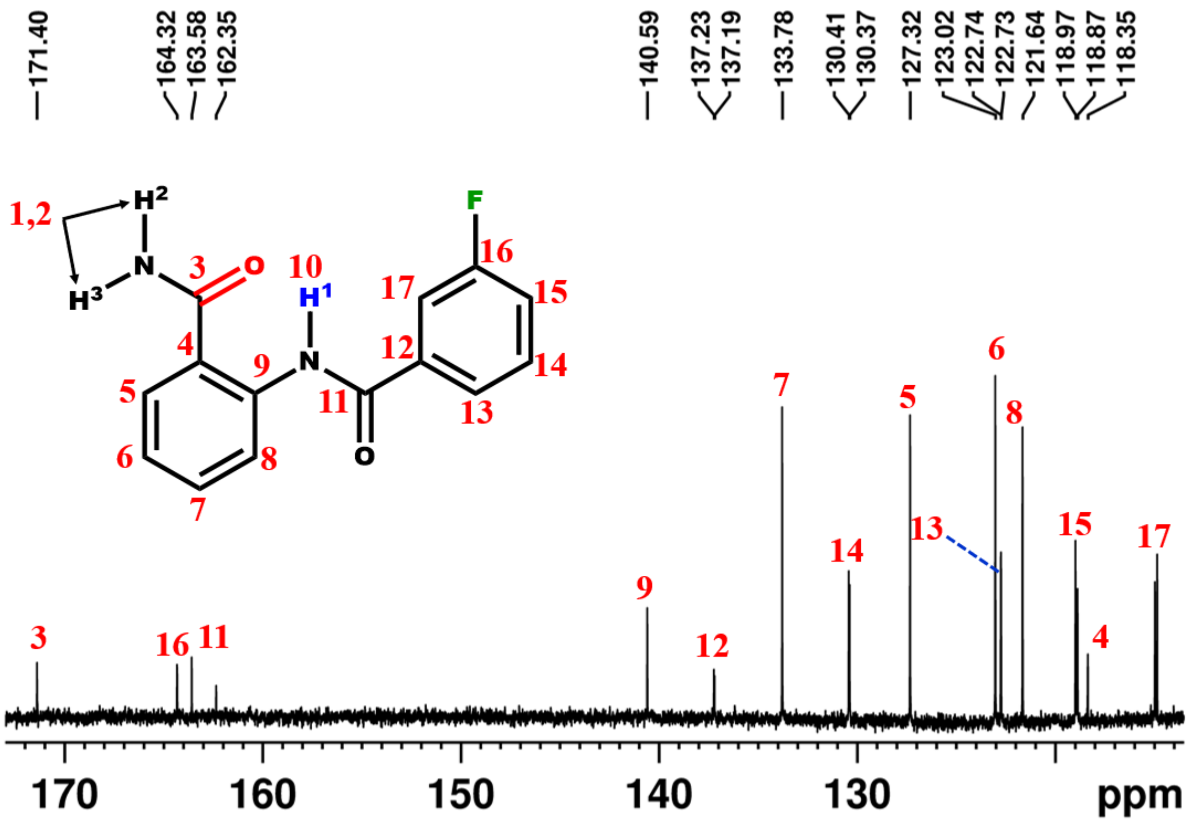

Figure S28: ^{13}C NMR spectrum of molecule **4**, acquired in 800 MHz spectrometer in the solvent $CDCl_3$ at 298K.

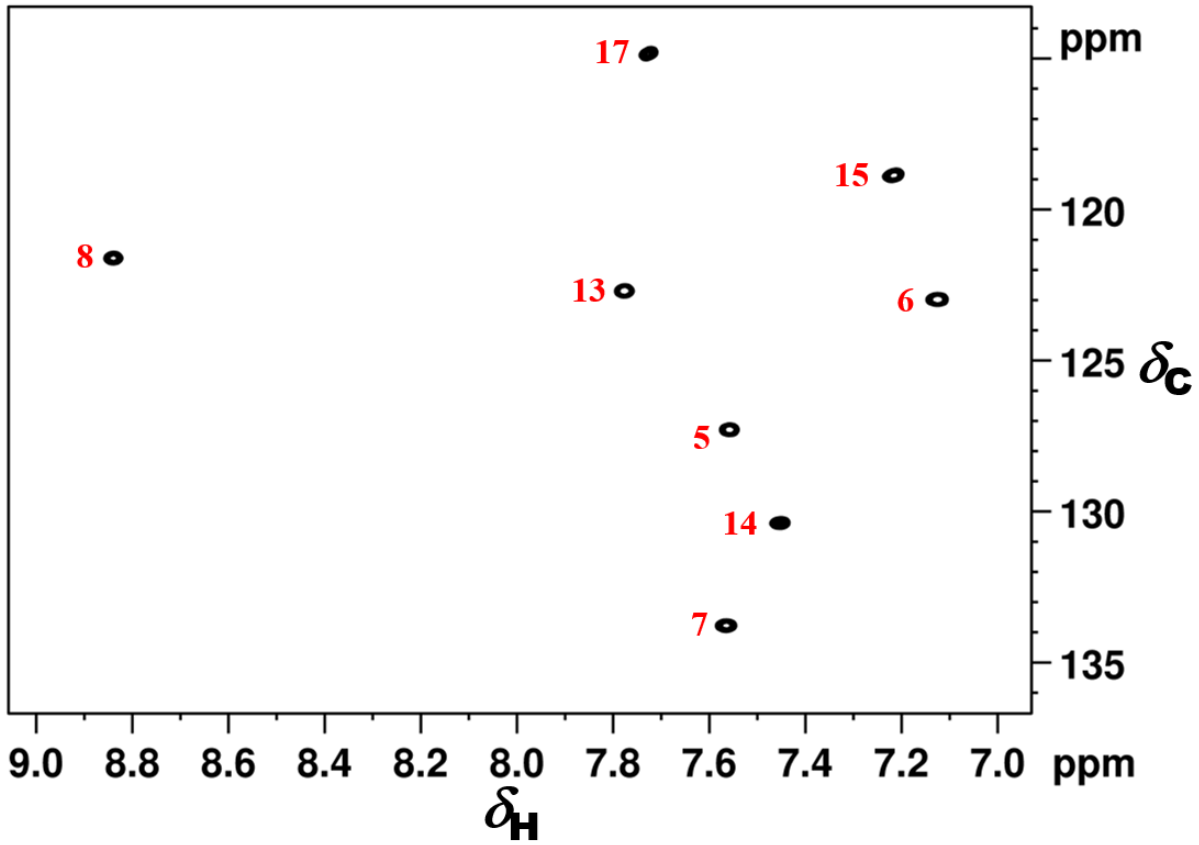

Figure S29: 2D ^{1}H-^{13}C HSQC NMR spectrum of molecule **4**, acquired in 800 MHz spectrometer in the solvent $CDCl_3$ at 298K.

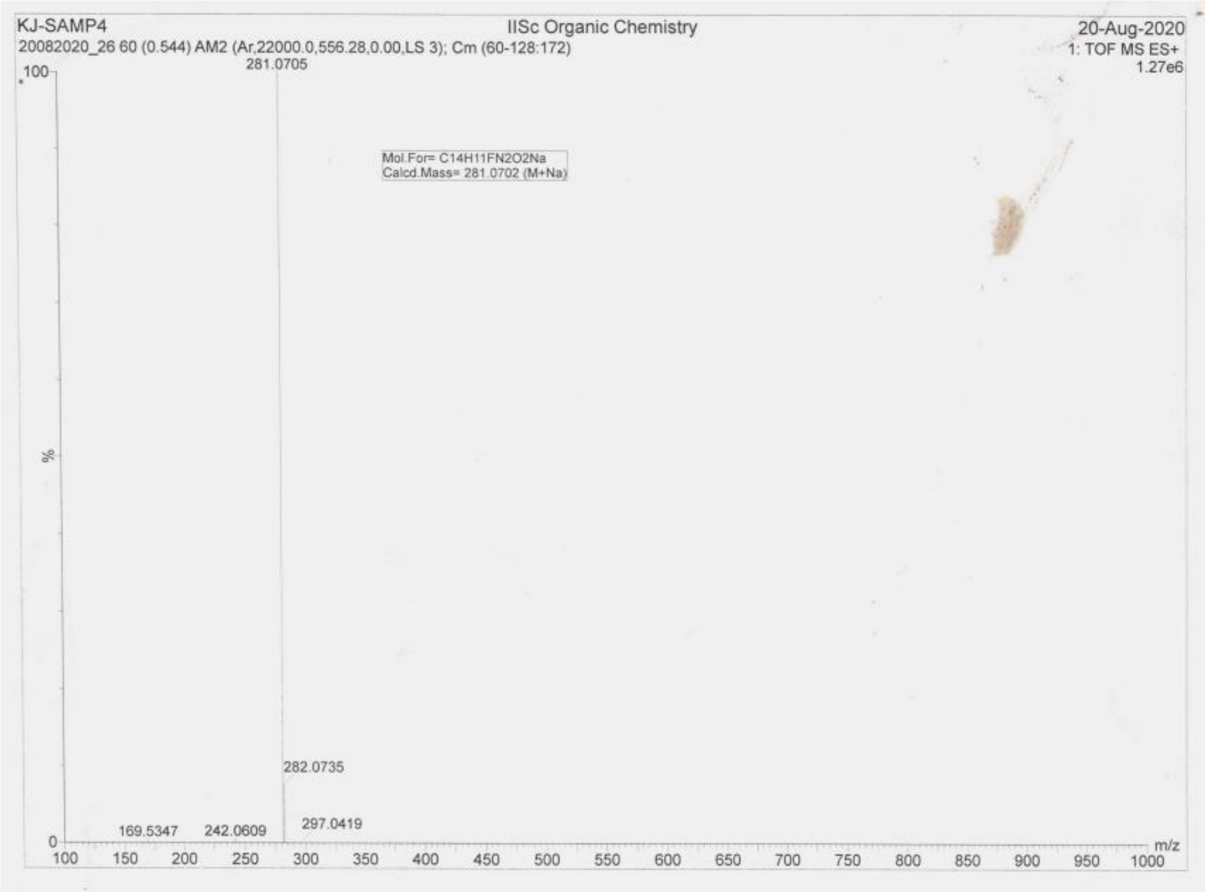

Figure S30: HRMS (ESI) $[M+Na]^+$ spectrum of molecule **4**.

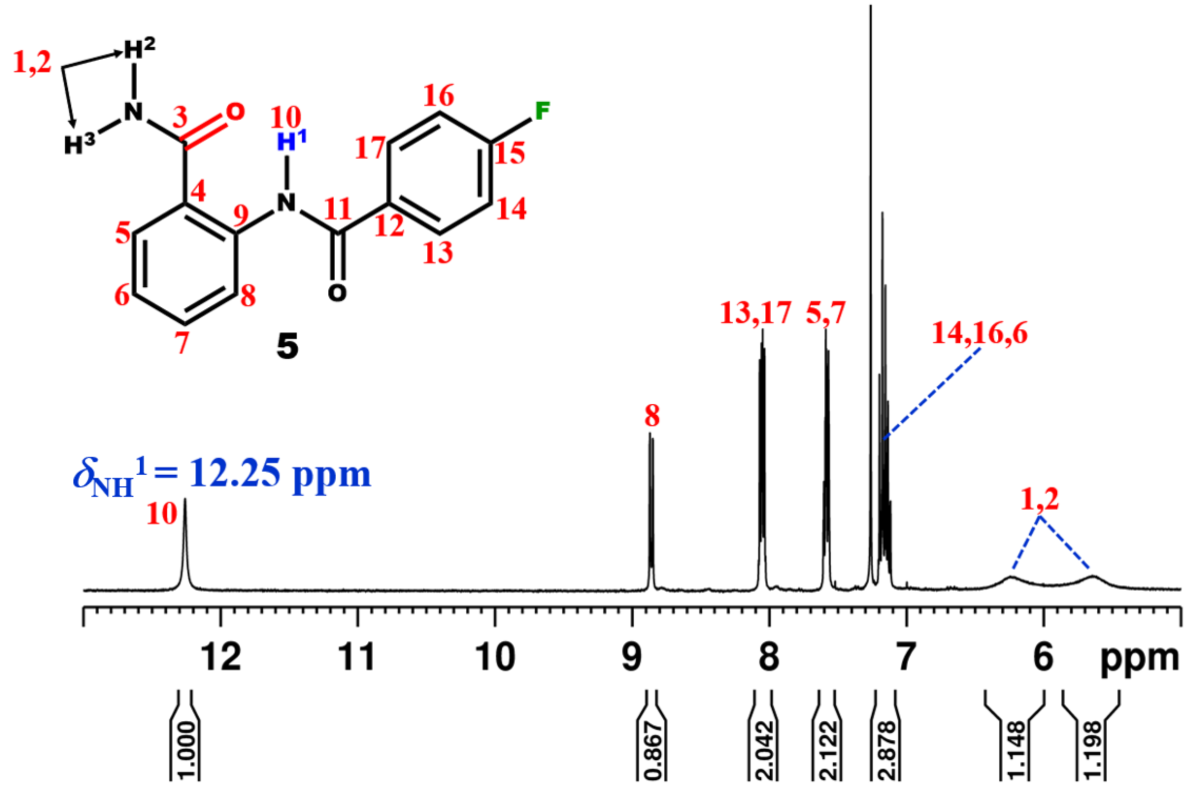

Figure S31: ^{1}H NMR spectrum of molecule **5**, acquired in 400 MHz spectrometer in the solvent $CDCl_3$ at 298K.

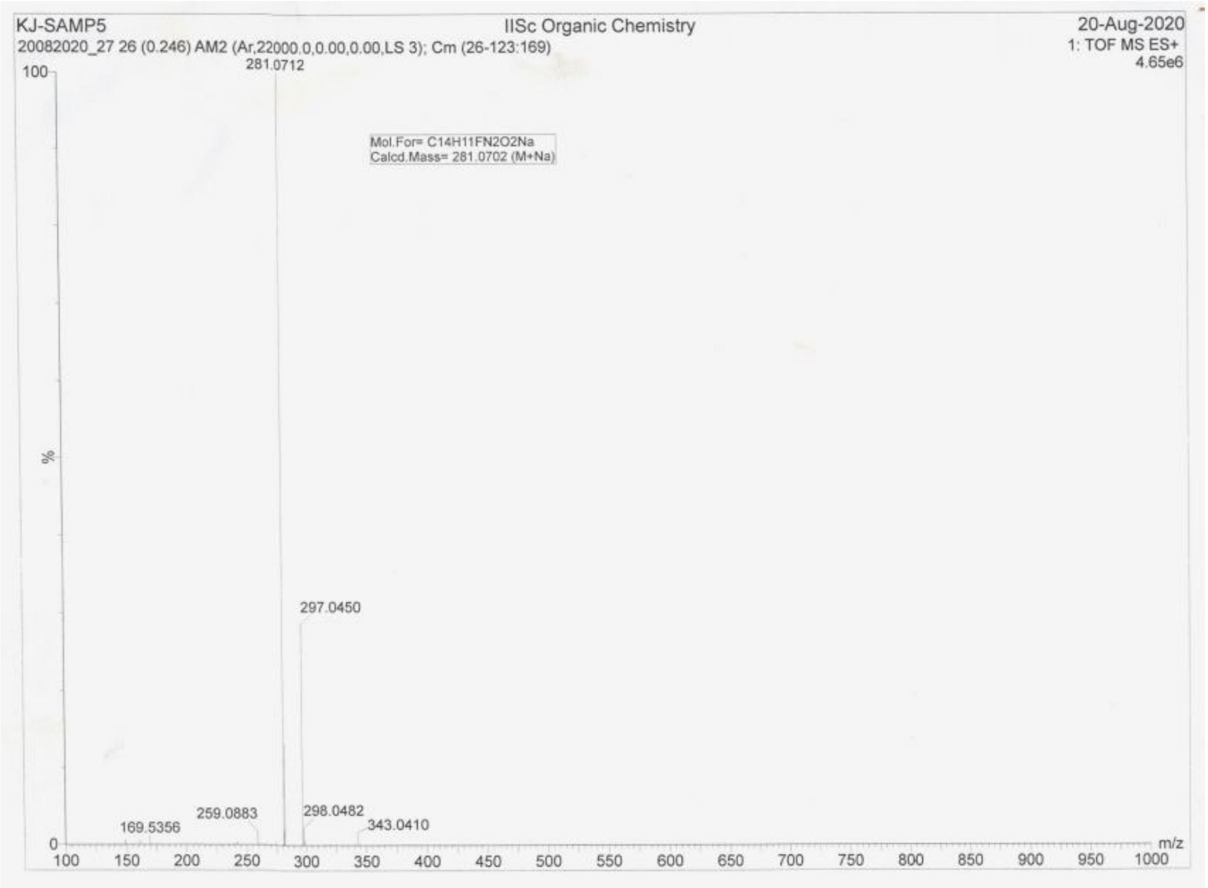

Figure S32: HRMS (ESI) [M+Na]$^{+}$ spectrum of molecule **5**.

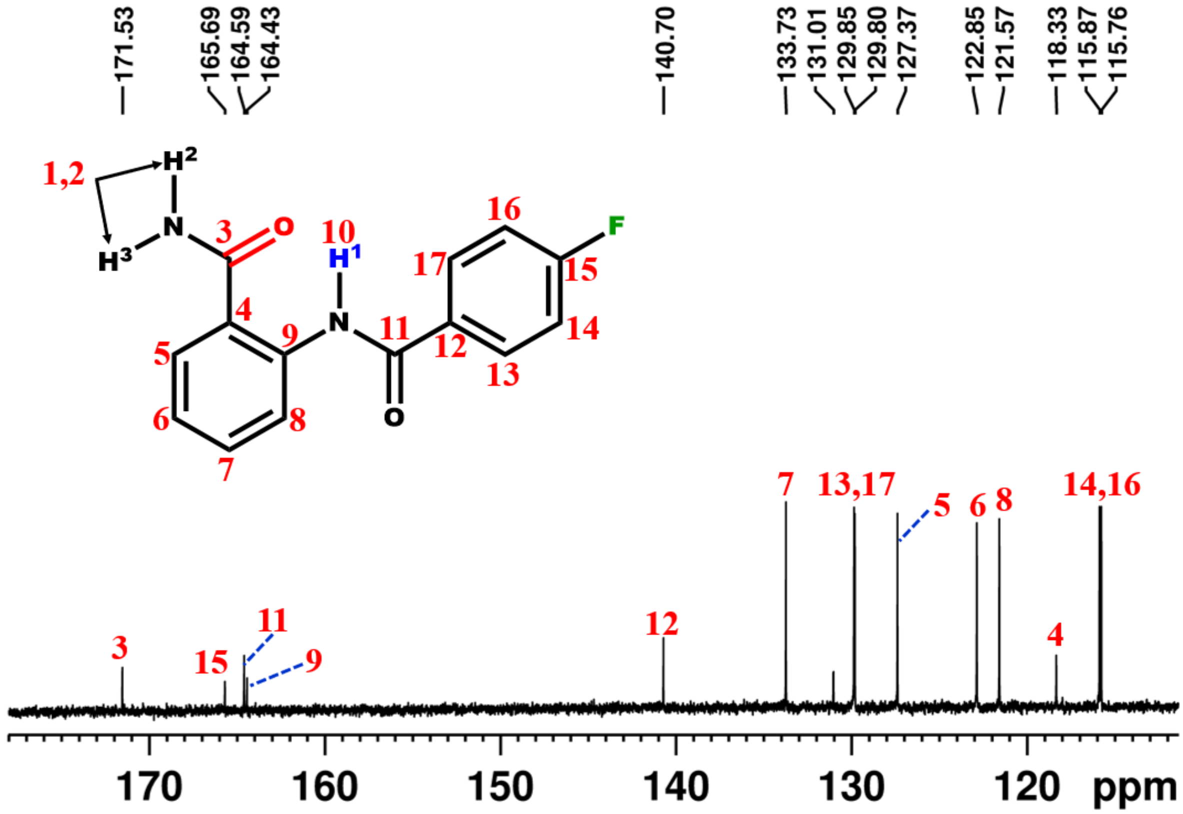

Figure S33: ^{13}C NMR spectrum of molecule **6**, acquired in 800 MHz spectrometer in the solvent $CDCl_3$ at 298K.

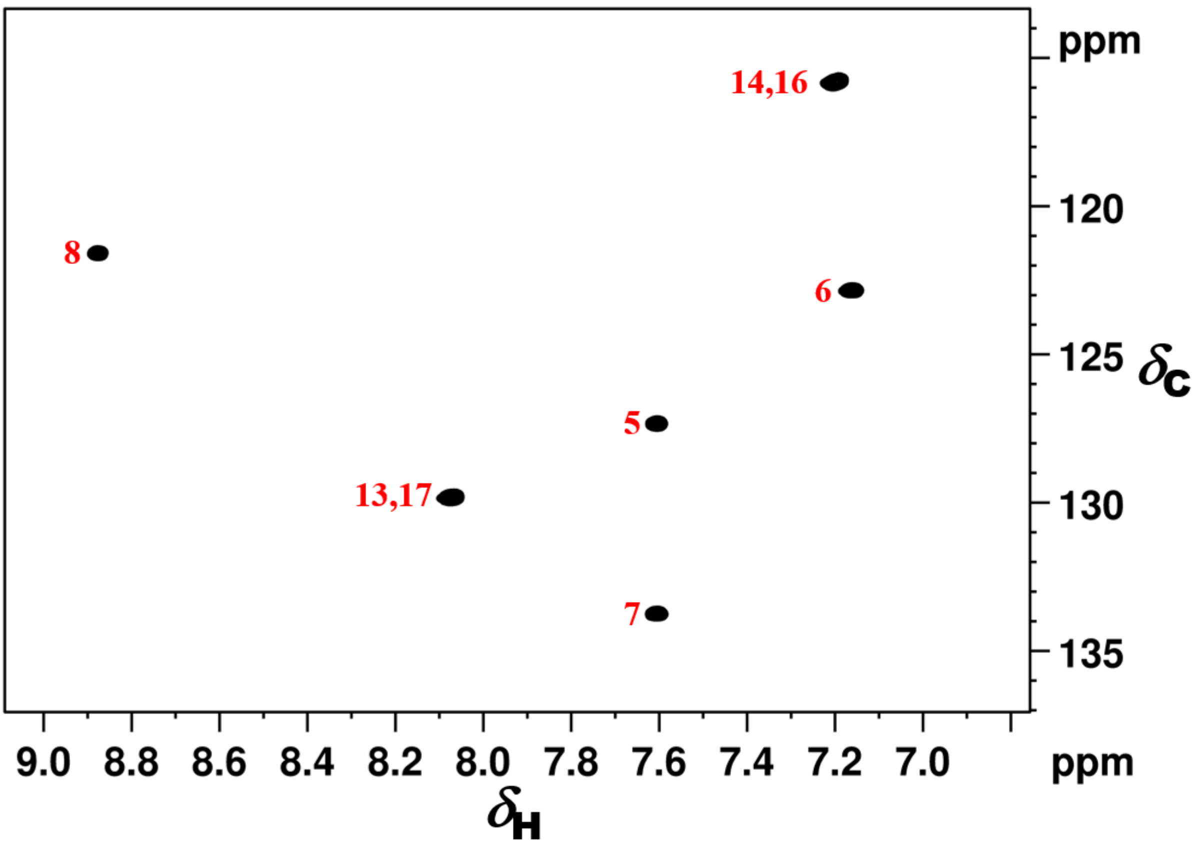

Figure S34: 2D ^{1}H-^{13}C HSQC NMR spectrum of molecule **6**, acquired in 800 MHz spectrometer in the solvent $CDCl_3$ at 298K.

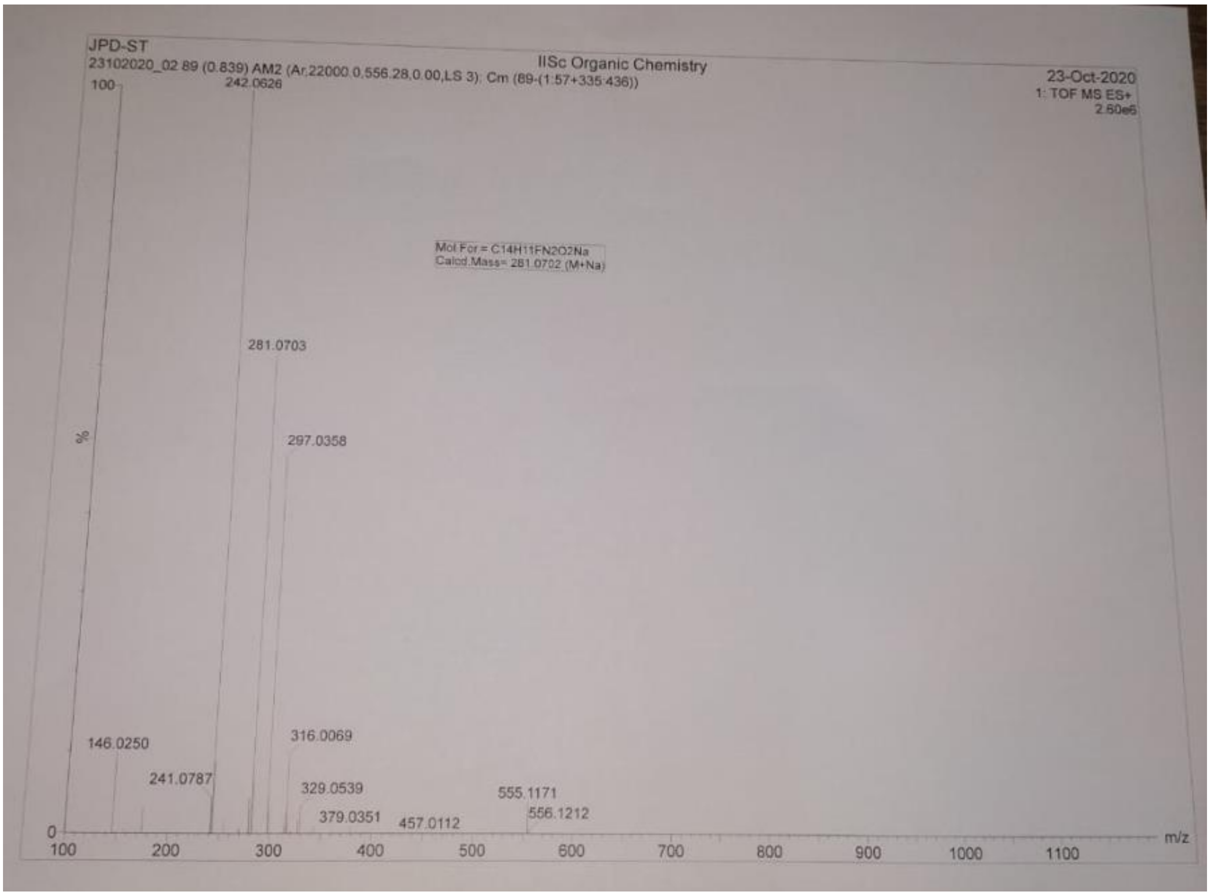

Figure S35: HRMS (ESI) [M+Na]$^+$ spectrum of molecule **6**.

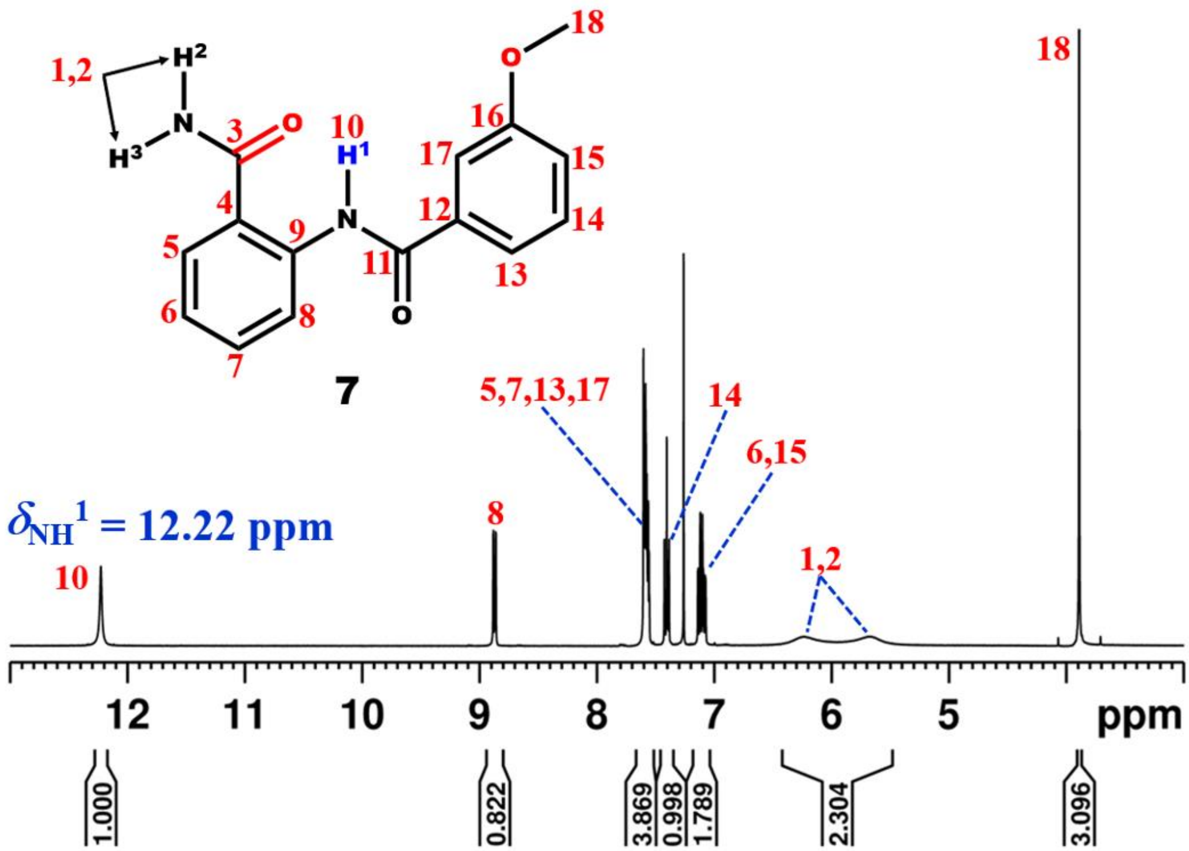

Figure S36: ^{1}H NMR spectrum of molecule **7**, acquired in 400 MHz spectrometer in the solvent $CDCl_3$ at 298K.

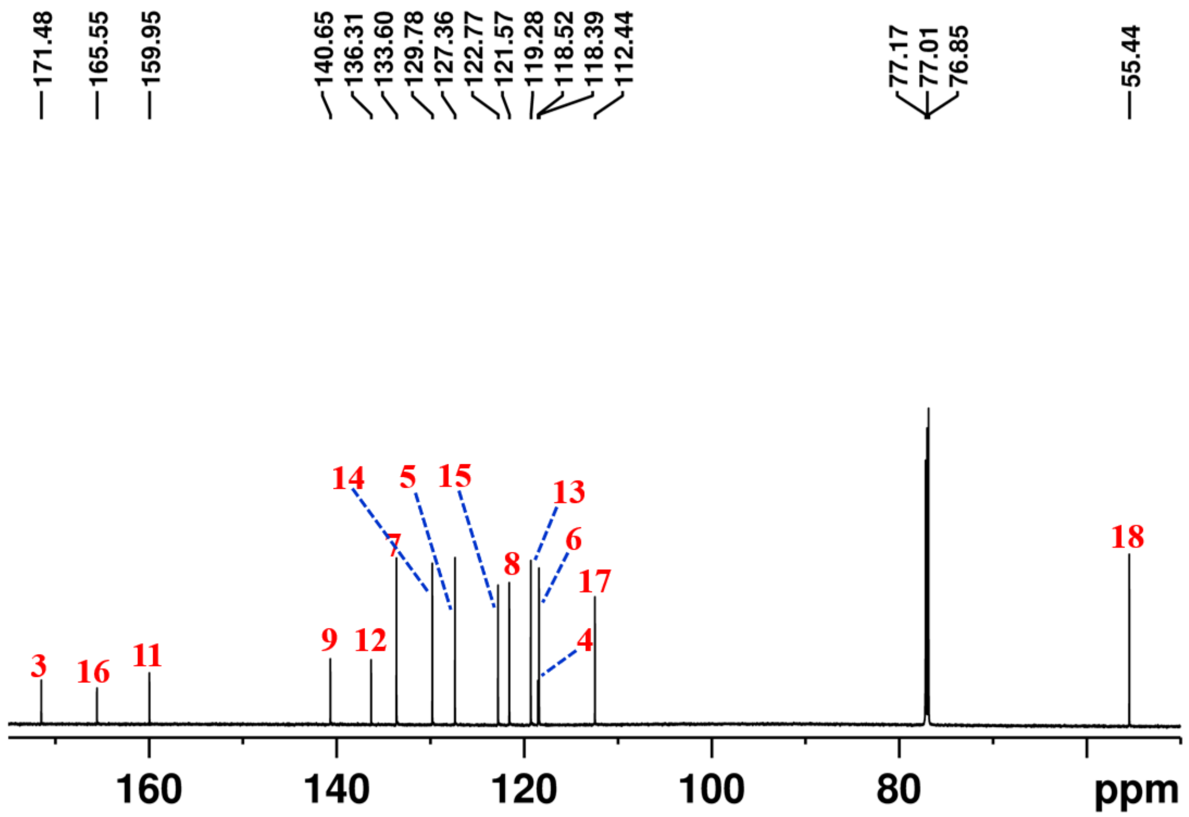

Figure S37: ^{13}C NMR spectrum of molecule **7**, acquired in 800 MHz spectrometer in the solvent $CDCl_3$ at 298K.

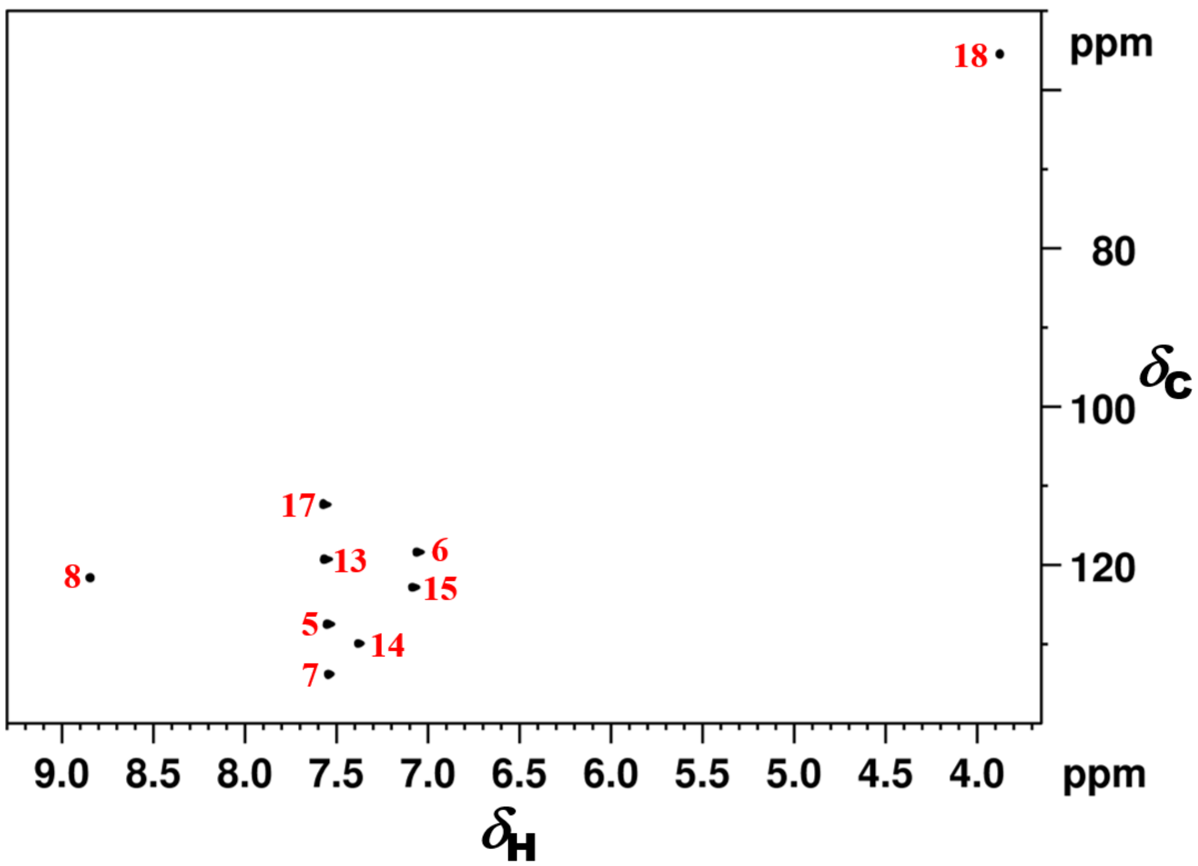

Figure S38: 2D ^{1}H-^{13}C HSQC NMR spectrum of molecule **7**, acquired in 800 MHz spectrometer in the solvent $CDCl_3$ at 298K.

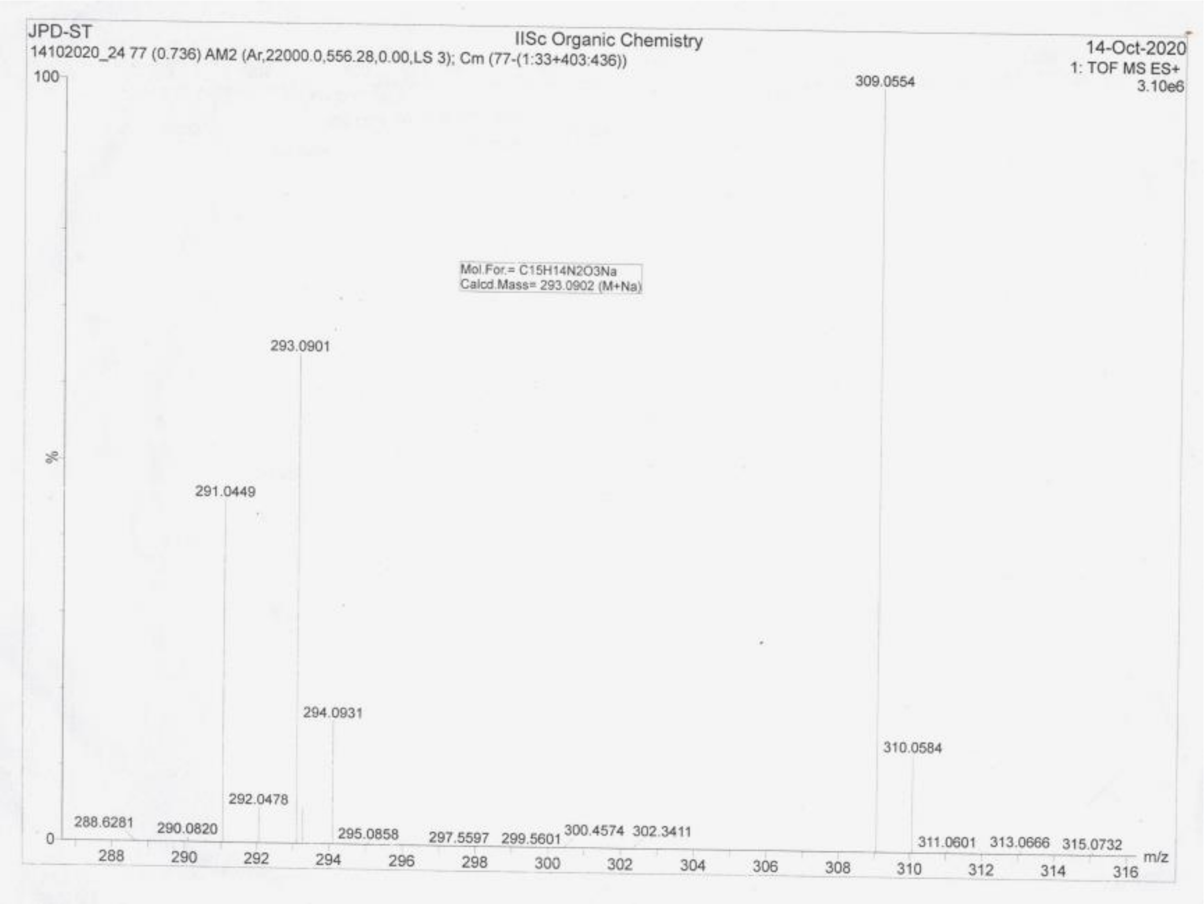

Figure S39: HRMS (ESI) $[M+Na]^+$ spectrum of molecule **7**.

Table S1: The amide temperature coefficients of molecules, **1-3**.

**Entry**	**Molecule**	**Amide temperature coefficients (ppb/K) of NH^1**
1	**1**	-0.3
2	**2**	-1
3	**3**	-1.3

Single Crystal X-Ray Diffraction (XRD) of Molecule 2

The single crystals of the molecule **2** (258.3 mg, 1 mmol) were obtained by dissolving the compound in 10 mL of chloroform and left undisturbed for slow evaporation at room temperature. After a few days of slow evaporation of the solvent, the colorless needle like crystals were obtained. The compound melted at 182-184^0 C. (CCDC Number: 2049223)

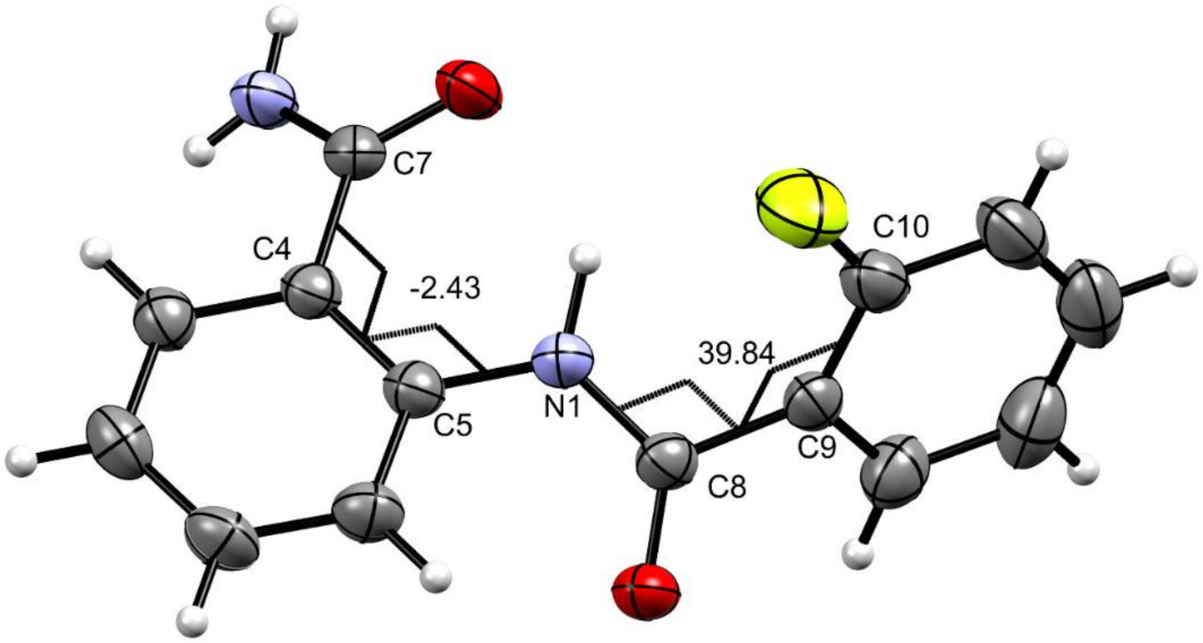

Table S2: Crystal data and structure refinement for molecule **2**.

Empirical formula	$C_{14} H_{11} F_1 N_2 O_2$	
Formula weight	258.25	
Temperature	296(2) K	
Wavelength	0.71073 Å	
Crystal system	Triclinic	
Space group	P -1	
Unit cell dimensions	a = 7.4017(4) Å	α = 97.121(2)°
	b = 9.1653(4) Å	β = 98.953(2)°
	c = 9.6034(4) Å	γ = 106.990(3)°
Volume	605.48(5) Å^3	
Z	2	
Density (calculated)	1.417 mg/m^3	
Absorption coefficient	0.107 mm^{-1}	

F(000)	268
Crystal size	0.3 x 0.2 x 0.1 mm^3
Theta range for data collection	2.362 to 27.549°.
Index ranges	-9<=h<=9, -11<=k<=11, -12<=l<=12
Reflections collected	10395
Independent reflections	2784 [R(int) = 0.0179]
Completeness to theta = 25.242°	99.8 %
Absorption correction	None
Refinement method	Full-matrix least-squares on F^2
Data / restraints / parameters	2784 / 0 / 184
Goodness-of-fit on F^2	1.036
Final R indices [I>2sigma(I)]	R1 = 0.0633, wR2 = 0.1959
R indices (all data)	R1 = 0.0683, wR2 = 0.2021
Extinction coefficient	n/a
Largest diff. peak and hole	1.528 and -0.501 e.$Å^{-3}$

DFT Optimized Structures

(a) (b)

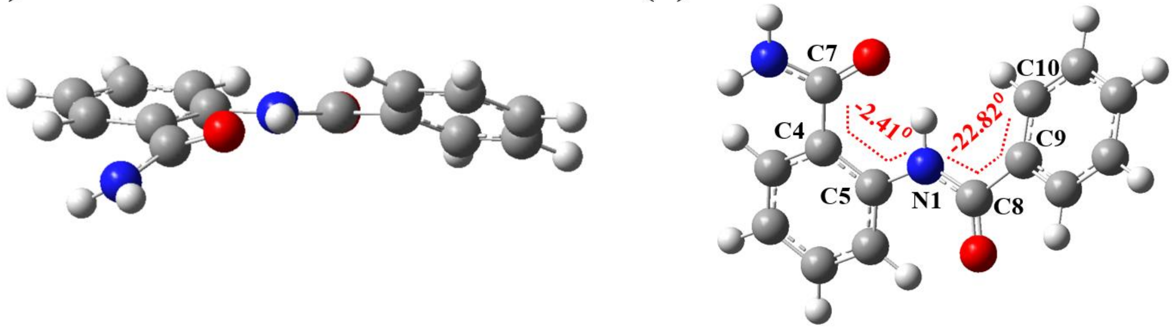

Figure S40: The DFT optimized spatial structure ((a) and (b) are two different projections) of molecule **1**.

(a) (b)

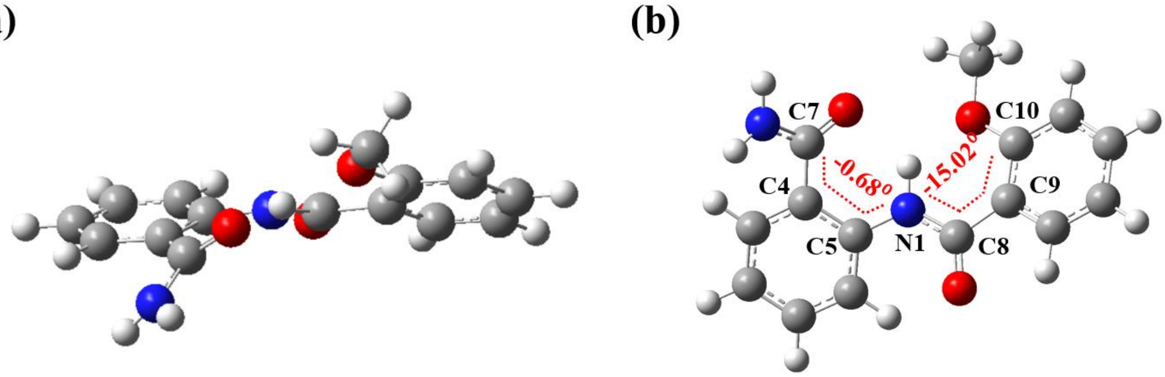

Figure S41: The DFT optimized spatial structure ((a) and (b) are two different projections) of molecule **3**.

(a) (b)

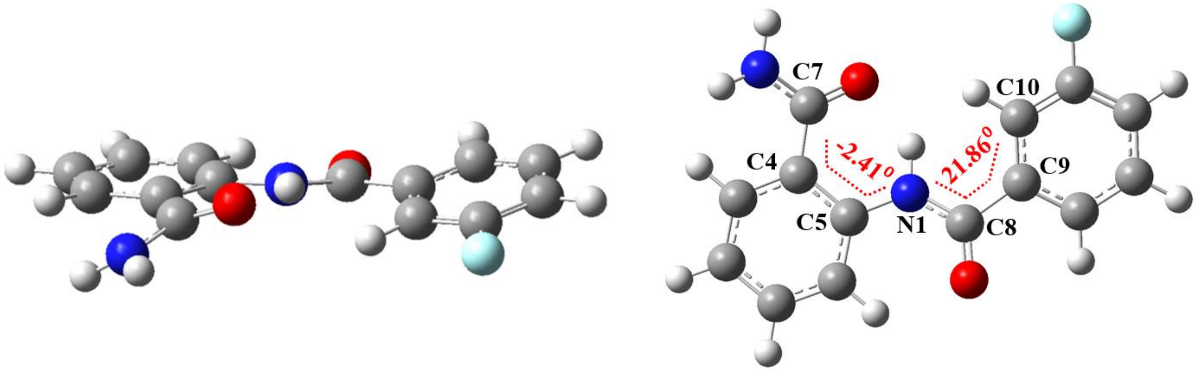

Figure S42: The DFT optimized spatial structure ((a) and (b) are two different projections) of molecule **4**.

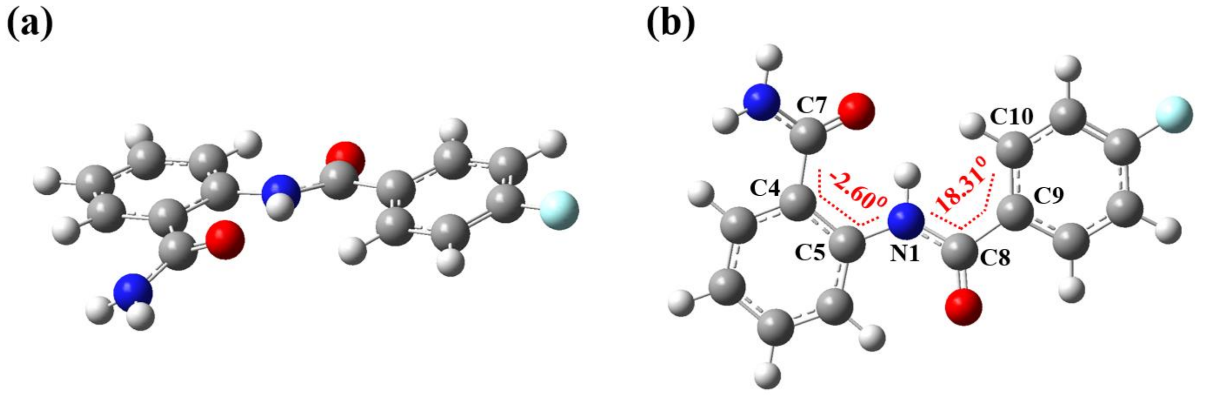

Figure S43: The DFT optimized spatial structure ((a) and (b) are two different projections) of molecule **5**.

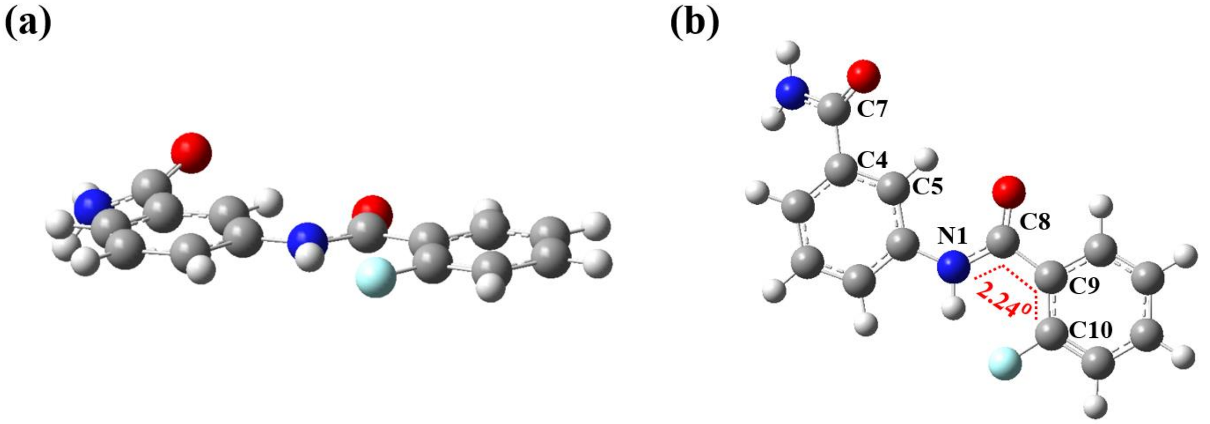

Figure S44: The DFT optimized spatial structure ((a) and (b) are two different projections) of molecule **6**.

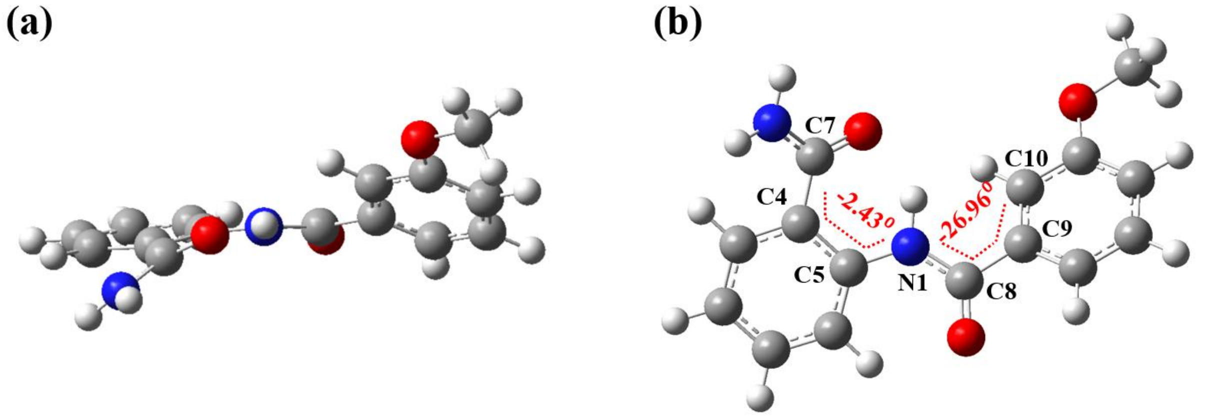

Figure S45: The DFT optimized spatial structure ((a) and (b) are two different projections) of molecule **7**.

Table S3: The comparision of theoretical chemical shift of NH[1] (δ_{NH1}) obtained from the DFT optimized spatial structure and the experimental values for molecules **1** to **7**.

Entry	Molecule	δ_{NH}^{1} (ppm)		$\Delta\delta_{NH}^{1}$ (ppm)
		**Theoretical values**	**Experimental values**	
1	**1**	12.59	12.22	0.37
2	**2**	12.09 ($^{1h}J_{FH}$= 1.04 Hz)	11.84 ($^{1h}J_{FH}$= 6.1 Hz)	0.25
3	**3**	11.94	11.83	0.11
4	**4**	12.74	12.23	0.51
5	**5**	12.67	12.25	0.42
6	**6**	9.11	8.57	0.54
7	**7**	13.11	12.22	0.89

$\Delta\delta_{NH}$ (ppm): Difference in the theoretical and experimental NMR chemical shift.

Experimental Section

All NMR spectra were recorded using Bruker AVANCE 400 MHz spectrometers. The TMS was used as internal reference for all the investigated molecules to measure the proton chemical shifts and all the spectra acquired at 298 K, except the variation temperature studies. The synthesized molecules were characterized by electron spray ionization mass spectrometry (ESI-HRMS) and various one- and two- dimensional NMR techniques. All the chemicals used for the synthesis were purchased from Sigma Aldrich and the deuterated solvents, such as, $CDCl_3$ and DMSO-d_6 were purchased from Cambridge Isotopes. XRD data were collected on a Bruker AXS Kappa Apex II CCD diffractometer with Mo K_α radiation. The structure was solved by direct methods using SHELXS97[8]and refined in the spherical atom approximation (based on F^2) by SHELXL97[8] using the WinGX suite[9].

General Synthesis of Molecules 1 to 7

The 1 equivalent of benzoyl chloride (500 mg, 3.67 mmol) of interest and pyridine (290.29 mg, 3.67 mmol) was added dropwise to the 1.09 equivalent of amino benzamide (4.003 mmol) of interest solution in 15 ml of chloroform at 0ºC. After that the ice bath is removed and the reaction mixture was stirred at room temperature for 1 hour. A precipitate obtained was filtered and washed with a copious amount of water. The trace of pyridine was evaporated by adding toluene solvent. The formation of N-benzoylanthranilamide and its derivatives was characterized by electron spray ionization mass spectrometry (ESI-HRMS) and using NMR techniques.

Molecule 1: Yield = 81 % (712 mg). White solid. **^{1}H-NMR ($CDCl_3$, 400 MHz, ppm, 298 K):** 12.22 (1H, s, NH), 8.89 (1H, dd, *J* = 8.77 Hz; 1.12 Hz, ArH), 8.02-8.05 (2H, m, ArH), 7.52-7.60 (5H, m, ArH), 7.13-7.14 (1H, m, ArH), 6.34 (1H, s, NH), and 5.73 (1H, s, NH). **^{13}C{^{1}H}-NMR ($CDCl_3$, 400 MHz, ppm, 298 K):** 171.51, 165.70, 140.77, 134.83, 133.70, 131.90, 128.77, 127.41, 127.32, 122.77, 121.67, and 118.44. **HRMS (ESI) [M+Na]$^+$:** m/z calculated for $C_{14}H_{12}N_2O_2Na$ 263.0796 and found 263.0798. **Melting Point:** 215-217 ˚C.

Molecule 2: Yield = 54 % (510 mg). Pink crystalline solid. **^{1}H-NMR ($CDCl_3$, 400 MHz, ppm, 298 K):** 11.84 (1H, d, *J* = 6.1 Hz, NH), 8.80 (1H, dd, *J* = 8.72 Hz; 1.09 Hz, ArH), 8.05 (1H, td, *J* = 7.75 Hz; 1.84 Hz, ArH), 7.47-7.58 (3H, m, ArH), 7.26-7.29 (1H, m, ArH), 7.12-7.21 (2H, s, ArH), 6.13 (1H, s, NH), and 5.65 (1H, s, NH). **^{13}C{^{1}H}-NMR ($CDCl_3$, 400 MHz, ppm, 298 K):** 171.12, 162.50, 161.11, 159.86, 139.73, 133.63, 133.59, 131.72, 127.45, 123.29,

122.54, 120.24, and 116.56. **HRMS (ESI) [M+Na]$^+$:** m/z calculated for $C_{14}H_{11}FN_2O_2Na$ 281.0702 and found 281.0703. **Melting Point:** 182-184 ˚C.

<u>**Molecule 3**</u>**: Yield =** 73 % (720 mg). White solid. **^{1}H-NMR ($CDCl_3$, 400 MHz, ppm, 298 K):** 11.83 (1H, s, NH), 8.72 (1H, d, *J* = 8.22 Hz, ArH), 8.20 (1H, dd, *J* = 7.84 Hz, 1.77 Hz; ArH), 7.45-7.54 (3H, m, ArH), 7.00-7.12 (3H, m, ArH), 6.02 (1H, s, NH), 5.57 (1H, s, NH), and 4.08 (3H, s, OCH_3). **^{13}C{^{1}H}-NMR ($CDCl_3$, 400 MHz, ppm, 298 K):** 170.77, 164.30, 157.74, 138.94, 133.19, 132.54, 131.36, 127.13, 123.40, 123.03, 122.34, 122.32, 120.96, 111.34 and 55.65. **HRMS (ESI) [M+Na]$^+$:** m/z calculated for $C_{15}H_{14}N_2O_3Na$ 293.0902 and found 293.0902**. Melting Point:** 220-222 ˚C.

<u>**Molecule 4**</u>**: Yield =** 71 % (680 mg). White solid. **^{1}H-NMR ($CDCl_3$, 400 MHz, ppm, 298 K):** 12.30 (1H, s, NH), 8.86 (1H, dd, *J* = 8.73 Hz; 0.98 Hz, ArH), 7.73-7.81 (2H, m, ArH), 7.57-7.60 (1H, m, ArH), 7.45-7.50 (1H, m, ArH), 7.21-7.25 (1H, s, ArH), 7.12-7.16 (1H, s, ArH), 6.22 (1H, s, NH), and 5.67 (1H, s, NH). **^{13}C{^{1}H}-NMR ($CDCl_3$, 400 MHz, ppm, 298 K):** 171.39, 164.32, 163.58, 140.58, 137.22, 133.77, 130.40, 127.32, 123.01, 122.74, 122.72, 121.64, 118.86, 114.96, and 114.84. **HRMS (ESI) [M+Na]$^+$:** m/z calculated for $C_{14}H_{11}FN_2O_2Na$ 281.0702 and found 281.0705**. Melting Point:** 197-200 ˚C.

<u>**Molecule 5**</u>**: Yield =** 85 % (810 mg). White solid. **^{1}H-NMR ($CDCl_3$, 400 MHz, ppm, 298 K):** 8.57 (1H, s, NH), 8.15-8.20 (1H, m, ArH), 7.88 (1H, m, ArH), 7.45-7.63 (3H, m, ArH), 7.32-7.36 (1H, m, ArH), 7.18-7.23 (1H, s, ArH), 6.18 (1H, s, NH), and 5.57 (1H, s, NH). Because of the solubility the other ^{13}C{^{1}H}-NMR could not be obtained. **HRMS (ESI) [M+Na]$^+$:** m/z calculated for $C_{14}H_{11}FN_2O_2Na$ 281.0702 and found 281.0703. **Melting Point:** 201-203 ˚C.

<u>**Molecule 6**</u>**: Yield =** 71 % (709 mg). **^{1}H-NMR ($CDCl_3$, 400 MHz, ppm, 298 K):** 12.28 (1H, s, NH), 8.86 (1H, d, *J* = 8.26 Hz, ArH), 8.06 (2H, m, ArH), 7.58-7.60 (2H, m, ArH), 7.18-7.20 (2H, m, ArH), 7.14-7.15 (1H, m, ArH), 6.33 (1H, s, NH), and 5.69 (1H, s, NH). **^{13}C{^{1}H}-NMR ($CDCl_3$, 400 MHz, ppm, 298 K):** 171.52, 165.68, 164.58, 164.42, 140.70, 133.72, 131.01, 129.84, 129.80, 127.37, 122.85, 121.56, 118.32, 115.86 and 115.75. **HRMS (ESI) [M+Na]$^+$:** m/z calculated for $C_{14}H_{11}FN_2O_2Na$ 281.0702 and found 281.0712. **Melting Point:** 216-218 ˚C.

<u>**Molecule 7**</u>**: Yield =** 75 % (715 mg). **^{1}H-NMR ($CDCl_3$, 400 MHz, ppm, 298 K):** 12.22 (1H, s, NH), 8.85 (1H, d, *J* = 8.16 Hz, ArH), 7.55-7.59 (4H, m, ArH), 7.39-7.41 (1H, m, ArH), 7.07-7.11 (2H, m, ArH), 6.33 (1H, s, NH), 5.71 (1H, s, NH), and 3.88 (3H, s, OCH_3). **^{13}C{^{1}H}-NMR ($CDCl_3$, 400 MHz, ppm, 298 K):** 171.47, 165.54, 159.94, 140.65, 136.30, 133.60, 129.77,

127.36, 122.77, 121.56, 119.27, 118.51, 118.39, 112.44 and 55.44. **HRMS (ESI) [M+Na]$^+$:** m/z calculated for $C_{15}H_{14}N_2O_3Na$ 293.0902 and found 293.0901. **Melting Point:** 176-179 ˚C.

References:

[1] A.K. Patel, S. K. Mishra, K. Krishnamurthy and N. Suryaprakash, ***RSC Adv.*** **2019**, *9*, 32759-32770.

[2] N. Arya, S. K. Mishra and N. Suryaprakash, *New J. Chem.* **2019**, *43*, 13134-13142.

[3] P. Dhanishta, S. K. Mishra and N. Suryaprakash, *J. Phys. Chem. A.* **2018**, *122*, 199-208.

[4] A. Lakshmipriya and N. Suryaprakash, *J. Phys. Chem. A* **2016**, *120*, 7810-7816.

[5] S.K. Mishra and N. Suryaprakash, *RSC Adv.* **2015**, *5*, 86013-86022.

[6] S. K. Mishra and N. Suryaprakash, *Phys. Chem. Chem. Phys.* **2015**, *17*, 15226-15235.

[7] S. R. Chaudhari, S. Mogurampelly, and N. Suryaprakash, *J. Phys. Chem. B* **2013**, *117*, 1123-1129.

[8] G. M. Sheldrick, *Acta Crystallogr. Sect. A: Found. Crystallogr.* **2008**, *64*, 112-122.

[9] L. J. Farrugia, *J. Appl. Crystallogr.* **1999**, *32*, 837-838.
